# Larvicidal Activity of Cinnamic Acid Derivatives: Investigating Alternative Products for *Aedes aegypti* L. Control

**DOI:** 10.3390/molecules26010061

**Published:** 2020-12-24

**Authors:** Marianna O. Araújo, Yunierkis Pérez-Castillo, Louise H. G. Oliveira, Fabíola C. Nunes, Damião P. de Sousa

**Affiliations:** 1 Post Graduation Program in Natural and Synthetic Bioactive Products, Federal University of Paraíba, João Pessoa 58051-900, Brazil; marianna.oliveira@ltf.ufpb.br; 2 Bio-Cheminformatics Research Group and Escuela de Ciencias Físicas y Matemáticas, Universidad de Las Américas, Quito 170125, Ecuador; yunierkis.perez@udla.edu.ec; 3 Biotechnology Center, Federal University of Paraíba, João Pessoa 58051-900, Brazil; louiseguimaraes@outlook.com (L.H.G.O.); fabiola@cbiotec.ufpb.br (F.C.N.); 4 Department of Pharmaceutical Sciences, Federal University of Paraíba, João Pessoa 58051-970, Brazil

**Keywords:** natural products, medicinal plants, mosquitoes, cinnamic ester, dengue, yellow fever, Zika, Chikungunya, larvicidal activity

## Abstract

The mosquito *Aedes aegypti* transmits the virus that causes dengue, yellow fever, Zika and Chikungunya viruses, and in several regions of the planet represents a vector of great clinical importance. In terms of mortality and morbidity, infections caused by *Ae. aegypti* are among the most serious arthropod transmitted viral diseases. The present study investigated the larvicidal potential of seventeen cinnamic acid derivatives against fourth stage *Ae. aegypti* larvae. The larvicide assays were performed using larval mortality rates to determine lethal concentration (LC_50_). Compounds containing the medium alkyl chains butyl cinnamate (**7**) and pentyl cinnamate (**8**) presented excellent larvicidal activity with LC_50_ values of around 0.21–0.17 mM, respectively. While among the derivatives with aryl substituents, the best LC_50_ result was 0.55 mM for benzyl cinnamate (**13**). The tested derivatives were natural compounds and in pharmacology and antiparasitic studies, many have been evaluated using biological models for environmental and toxicological safety. Molecular modeling analyses suggest that the larvicidal activity of these compounds might be due to a multi-target mechanism of action involving inhibition of a carbonic anhydrase (CA), a histone deacetylase (HDAC2), and two sodium-dependent cation-chloride co-transporters (CCC2 e CCC3).

## 1. Introduction

Arbovirus infections are caused by viruses transmitted to people through the bite of an infected arthropod, such as the mosquito. Dengue, yellow fever, Zika and Chikungunya are all transmitted by *Aedes aegypti* L., which is of great epidemiological importance as the main vector of arboviral infections in tropical and subtropical regions [1,2]. Of the above-mentioned diseases, dengue is the most prevalent, being present on five continents, and affecting about 50 million people per year around the world [3]. Dengue presents great potential to assume severe and lethal forms [4].

As current antiviral drugs are non-specific, and effective vaccines are lacking, effective strategies to combat such diseases are needed. One of the most effective preventative measures is vector control through insecticides [5]. However, indiscriminate use of insecticides often damages the environment and the occurrence of the resistant mosquito is now frequent [6]. An alternative is the search for natural insecticides to control mosquito populations, as they are generally highly biodegradable. For example, *Schinus terebinthifolius* leaf extract showed larvicidal activity in *Ae. aegypti* via lesion of the midgut, interfering with the survival and development of the larval phase. Derivatives of cinnamic acid should contribute to the larvicidal effect of the extract [7]. Now, rather than killing the adult mosquito, researchers are concentrating on synthetic compounds that kill the larvae at an initial stage [8].

Cinnamic acid is a low toxicity phenylpropanoid present in fruits, and extensively distributed in plants [9,10]. Certain cinnamic acid derivatives are found in plants as secondary metabolites, such as ethyl cinnamate in the *Kaempferia galanga* L. rhizome [11,12]; isopropyl cinnamate in *Kaempferia galanga* L. [13]; butyl cinnamate in *Mandragora autumnalis* Bertol. [14,15]; and pentyl cinnamate, a natural ester found in essential oil from the aerial parts of *Piper pierrei* C. DC. [16]. Benzyl cinnamate is found in essential oils of *Trachyspermum ammi* L. and *Myroxylon pereira* K. [17]. Various secondary metabolites are produced by plants for protection against predator insects. These are natural candidates in the search for new products to combat *Ae. aegypti* [18].

Various studies have shown that cinnamic acid derivatives present antioxidant [10], hypoglycemic [19,20], anti-dyslipidemic [21], antimicrobial [22], hepatoprotective [23], antimalarial [24] and larvicidal potential [25]. For example, Fujiwara and contributors (2017) [26] have already demonstrated the excellent larvicidal activity of methyl cinnamate against *Ae. aegypti*. Therefore, the purpose of the study was to investigate the larvicidal activity and the possible modes of action of cinnamic acid derivatives against *Ae. aegypti* L. larvae aiming to contribute to the development of new and safer larvicidal agents against disease-transmitting mosquitoes.

## 2. Results and Discussion

### 2.1. Effect of Cinnamic Acid Derivatives on Ae. aegypti Larvae

**Scheme 1:**
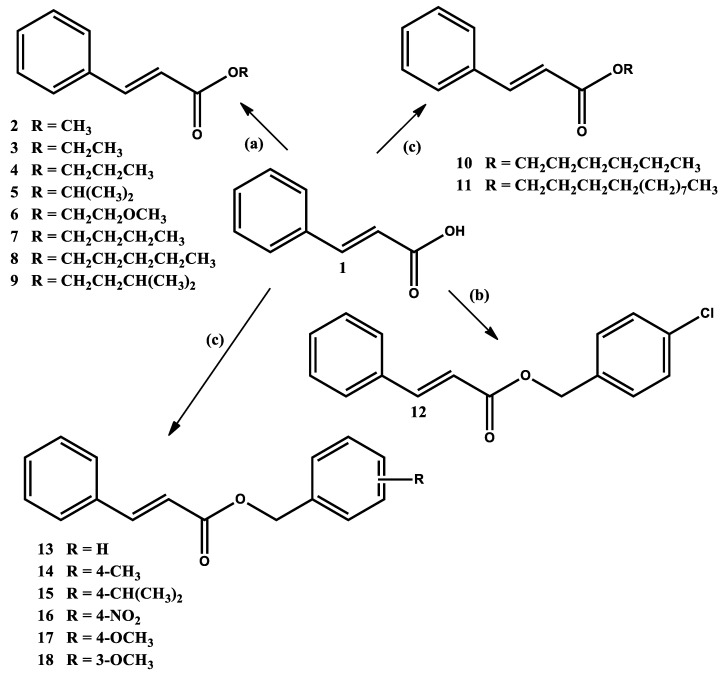
Synthesis of the cinnamic acid derivatives: (**a**) ROH, H_2_SO_4_, reflux, (**b**) Et_3_N, RX, acetone, reflux, (**c**) ROH, THF, TPP, DIAD, 0 °C at room temperature.

Cinnamic acid exhibits larvicidal activity, and it was used as a starting material to obtain 17 potentially bioactive derivatives, presented in Scheme 1. In this comparative study, the compounds were evaluated for their in vitro larvicidal activity against L4 larvae of *Ae. aegypti* L. The results are expressed as median lethal concentration (LC_50_) in mM, shown in Table 1. 

Table 1 summarizes the median lethal concentration of the test compounds against *Ae. aegypti* larvae. Structure–activity relationship (SAR) analysis was performed to verify chemical structure characteristics that influences bioactivity.

**Figure 1:**
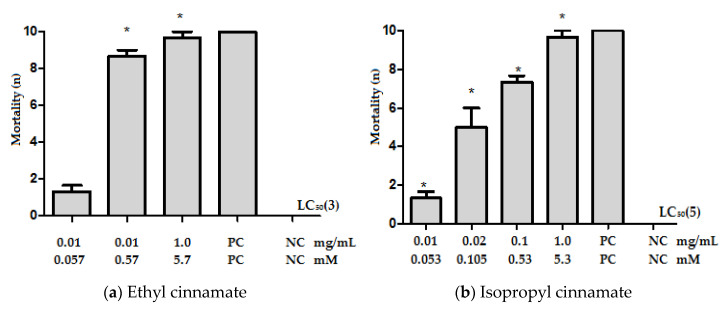

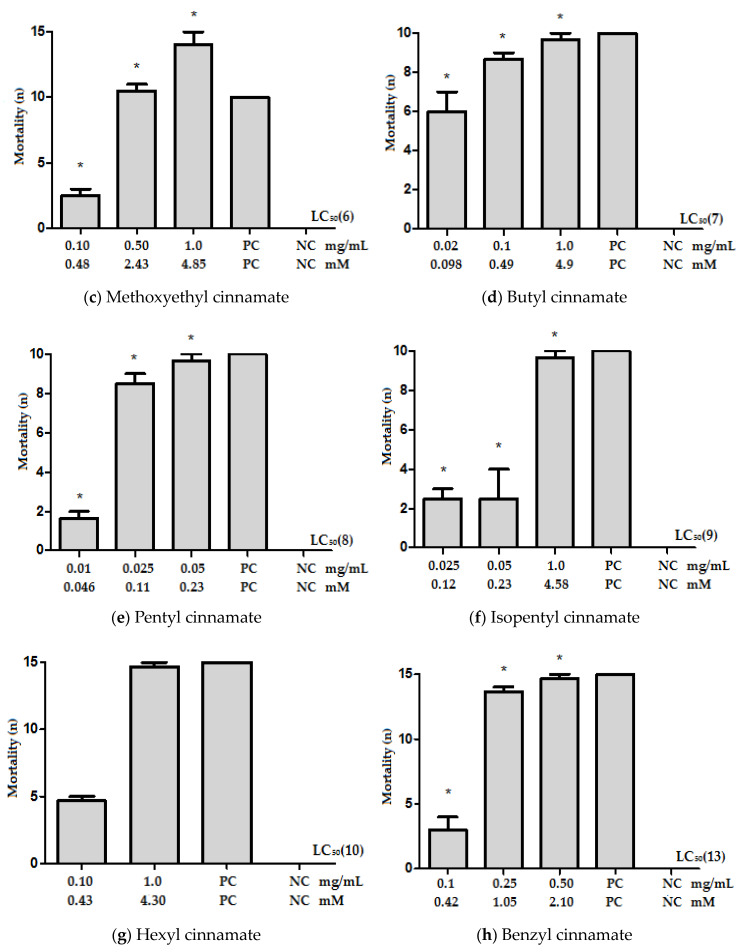

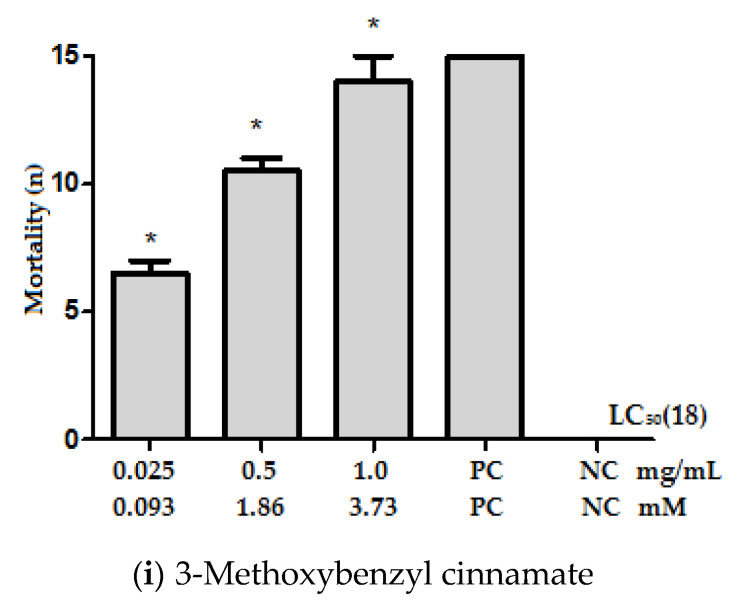
The best larvicidal activity among different cinnamic acid derivative concentrations: (**a**) ethyl cinnamate, (**b**) isopropyl cinnamate, (**c**) methoxyethyl cinnamate, (**d**) butyl cinnamate, (**e**) pentyl cinnamate, (**f**) isopentyl cinnamate, (**g**) hexyl cinnamate, (**h**) benzyl cinnamate and (**i**) 3-metoxybenzyl cinnamate against *Ae. aegypti* larvae after 24 h (expressed in mg/mL and convert mM). PC = positive control, NC = negative control. (*) Indicates results that are significantly different from the controls.

Most of the derivatives tested (except compounds **14**, **15**, **16** and **17**) demonstrated greater larvicidal potency than cinnamic acid (**1**) the starting material. The derivatives with alky substituents (compounds **2** through **11**) presented LC_50_ values ranging from 0.17 to 3.54 mM (*p* < 0.05). Methyl cinnamate (**2**) presented high potency compared to cinnamic acid (**1**); and ethyl cinnamate (**3**) with an ethyl group in its carbon chain presented increased larvicidal effect (LC_50_: 0.74 mM). However, these results are at odds with data from the literature in which it is reported that the presence of one or more alkyl groups in the chemical structure of cinnamic acid ester radicals lowers the intensity of larvicidal activity [27,28]. Certain compounds presented stronger larvicidal action, such as isopropyl cinnamate (**5**), methoxyethyl cinnamate (**6**), butyl cinnamate (**7**), pentyl cinnamate (**8**), isopentyl cinnamate (**9**) and hexyl cinnamate (**10**) (LC_50_: 0.52, 0.58, 0.21, 0.17, 0.83 and 0.99 mM, as shown respectively in Figure 1. These compounds present medium substituted alkyl chains such as compounds **7**, **8**, and **10**, a heteroatom in the carbon chain (compound **6**), and small to median carbon chain, for example compounds **5** and **9**; being the pentyl cinnamate (**8**) (the most active of all the derivatives tested) presented an LC_50_ of less than 0.2 mM. 

The less potent compounds (though better than the starting material), presented larger alkyl chains, for example dodecyl cinnamate (**11**); small alkyl chains such as methyl cinnamate (**2**) and propyl cinnamate (**4**).

The compounds presenting an aryl radical (compounds **12** to **18**) also presented less larvicidal effect than the alkyl cinnamates (compounds **2** to **11**); of these compounds, benzyl cinnamate (**13**), 4-chlorobenzyl cinnamate (**12**), and 3-metoxylbenzyl cinnamate (**18**) all presented better larvicidal activity than the starting material (**1**), as shown Figure 1. Compound **13** was the most potent aromatic derivative, with LC_50_: 0.55 mM [17]; yet the presence of ring substituents resulted in decreased larvicidal potency. Comparing compounds **14** and **15** suggests that both bulky alkyl substituents or non-bulky substituents attached to the aromatic ring in *para* position decrease larvicidal potency (LC_50_: 12.06 and 11.21 mM, respectively). The isomers **17** and **18**, present a methoxy substituent in different positions of the benzyl ring, respectively *para* or *meta*. Biological tests revealed that changing the position of this substituent for compound 18 increased larvicidal potency (LC_50_: 0.82 mM, Figure 1). Among the other aryl derivatives with electron withdrawing substituents, compounds **12** and **16**, only compound **12** showed greater larvicidal activity than the starting material (**1**), (LC_50_: 3.01 mM, Figure 1).

**Figure 2:**
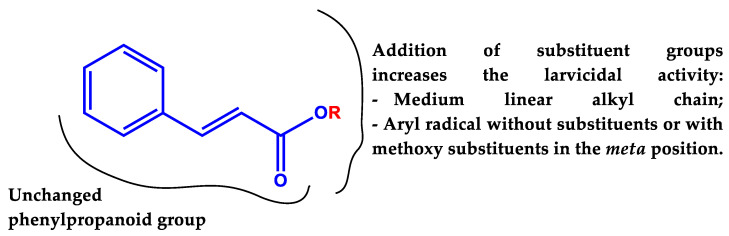
Structure–activity relationship (SAR) of cinnamic acid derivatives for larvicidal activity.

Thus, the SAR analysis shows certain structural features do increase larvicidal activity, such as cinnamate alkyl side chain, (especially the median linear carbon chain); or aryl radicals without substituents; or aryl radicals with *meta* position methoxy substituents. Figure 2 summarizes the study of cinnamic acid derivatives for larvicidal activity. The results are encouraging, and they show that esters of cinnamic acid are structurally simpler than the insecticide temephos, exhibit larvicidal potential, and are either found in nature or analogous to natural cinnamic derivatives.

### 2.2. Computational Methods

To study the potential mechanism of action of the compounds herein presented, modeling studies were performed as described in the Section 3. The list of the potential targets of the studied compounds is presented in Table 2 and it includes the UniProt [29] ID of the identified targets, the proteins descriptions, the genes ID of the target in the VectorBase [30] database and the ID assigned to each potential receptor throughout the rest of this report. Among the proteins listed in Table 2, GSTE2, GSTE4, GSTD2, GSTT1, GSTX2, GSTT2 and ACE were not predicted during the computational target fishing calculations. Regarding the Glutathione transferase enzymes, these were included in our study because there is evidence suggesting that cinnamic acid can inhibit their activity [31]. ACE was included because, despite there is no evidence pointing to its inhibition by cinnamic acid and its derivatives, it is the most widely studied target of insecticidal compounds in *Ae. aegypti* [32,33,34].

Molecular docking of compound **8** to the 27 receptors listed in Table 2 was performed as described in the Materials and Methods section. The detailed results of this step are provided in Table 3. Docking scores provided by Gold are dimensionless and the higher a score value is, the better the predicted pose is. The visual inspection of the predicted ligand-receptor complexes reveals meaningful complexes showing favorable ligand-receptor interactions and complementarity. This is further supported by the obtained docking scores. Following the same consensus scoring protocol used to select the probable binding modes of compound **8** to its potential receptors, it can be seen that the best consensus docking scores are obtained for the ACE, LGUL, HDAC2, RAC1 and GSTE2 targets. On the other hand, the CCC3, GLUR, GSTT1 and AKR6 targets are the ones with the poorest molecular docking scores. It is also worth noting that more than one possible binding mode is predicted for 22 out of the 27 proteins studied, leading to 60 compound **8**-receptor complexes to be analyzed.

**Figure 3:**
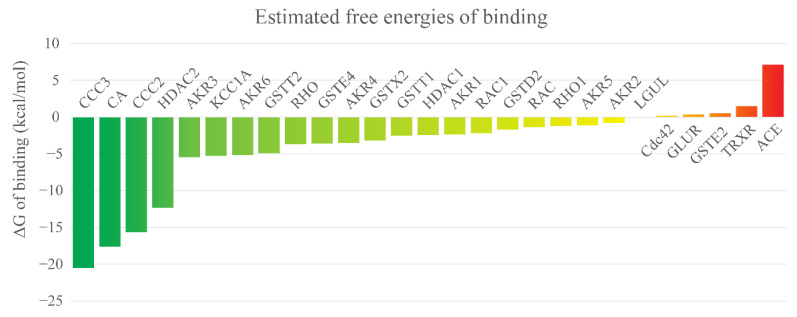
Predicted free energies of binding of compound **8** to its potential targets. The color scale goes from best (green) to worst (red) ΔGs of binding.

Due to the computational complexity of molecular recognition and the need of algorithms capable of processing large amounts of compounds in a reasonable time, many factors relevant for this process are simplified or neglected in molecular docking. It has also been shown that the estimation of the free energy of binding from Molecular Dynamics (MD) simulations snapshots during the post processing of molecular docking models can lead to a better selection of molecular targets of bioactive compounds [35,36]. Taking this into account, MD simulations and Molecular Mechanics-Poisson-Boltzmann Surface Area (MM-PBSA) calculations were performed for the 60 predicted compound **8**-receptor complexes as described in the Materials and Methods section. The detailed results of these calculations are provided in Table 4. The results of the MM-PBSA calculations are summarized in Figure 3.

The energetic and conformational stability of the complexes predicted for each target were analyzed. The plots of the total energy of the systems and of the ligand Root-Mean-Squared Deviation (RMSD) along the 20 production runs performed for each of these complexes are provided as Supplementary Materials in Figures S1 and S2, respectively. The RMSD calculations took place for the ligand, taking as reference its docking predicted pose. Overall, all systems are energetically stable during the production runs. Moreover, the ligand RMSD relative to the reference structure fluctuates around 2 Å or less for all complexes. These RMSD fluctuations are evidence of the conformational changes on the ligand bound to the receptors, indicating that diverse conformations are obtained for MM-PBSA calculations. 

The predicted free energies of binding show that the complexes with ACE, TRXE, GSTE2, GLUR and Cdc42 are predicted as unfeasible due to their positive ΔG values. ACE was not predicted by the target fishing approach and there is no evidence that it could be the target of the compounds herein studied. It was included in our investigations because it is the most widely studied target of insecticidal compounds. The fact that the worst (highest) ΔG value among all targets is obtained for ACE is consistent with these observations. Notably, the ACE and GSTE2 receptors that were among the top scored ones according to the molecular docking calculations are discarded as potential targets of compound **8** after the MM-PBSA calculations. These results highlight the importance of evaluating molecular complexes derived from docking calculations with more accurate methods such as MM-PBSA for the identification of potential targets for chemical compounds.

Compound **8** is predicted to have very low affinity for a group of proteins that includes LGUL, AKR2, AKR5, RHO1, RAC, GSTD2, RAC1. AKR1, HDAC1 and GSTT1 with predicted ΔG of binding ranging from −0.07 to −2.51 kcal/mol. A third set of targets formed by GSTX2, AKR4, GSTE4, RHO, GSTT2, AKR6, KCC1A and AKR3 is predicted with low to moderate ΔGs of binding. Interestingly, this set is enriched with proteins annotated with aldo-keto reductase and glutathione transferase functions, both involved in maintaining the redox equilibrium in the cell. Finally, the best values of predicted ΔG of binding are obtained for the HDAC2, CCC2, CA and CCC3 targets. 

**Figure 4:**
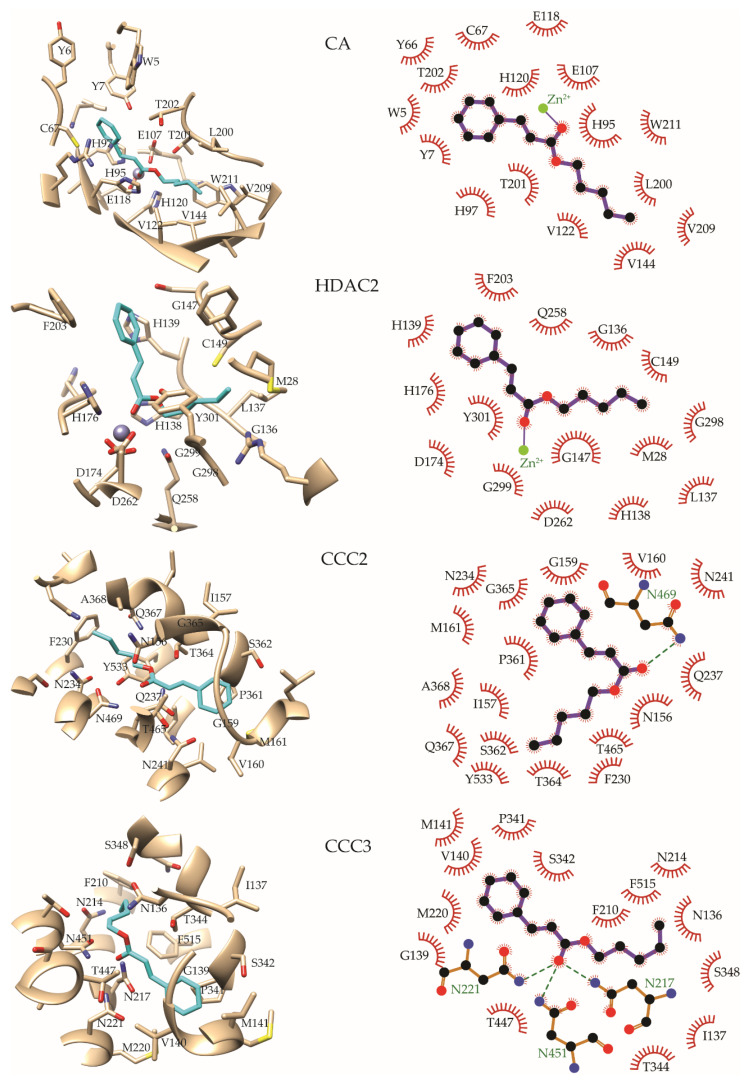
Predicted binding modes of compound **8** to CA, HDAC2, CCC2 and CCC3 (left) and the observed network of interactions with each target along the 200 MD snapshots used for Molecular Mechanics-Poisson-Boltzmann Surface Area (MM-PBSA) calculations (right). The receptors are depicted in gray and the ligand in cyan. Atoms are colored following the scheme: red for oxygen, blue for nitrogen and yellow for sulfur. Only residues interacting with the ligand on more than 50% of the analyzed MD snapshots are labelled. The Zn^2+^ ions at the active sites of CA and HDAC2 are represented as purple spheres. In the interaction diagrams, all atoms are represented only for amino acids forming hydrogen bonds with the ligand. In these, hydrogen bonds are represented by dashed lines, carbon atoms are represented in black and the same color scheme as in the left pictures is followed for the rest of the ligand atom types.

Overall, the obtained free energies of binding point to HDAC2, CCC2, CA and CCC3 as the most probable targets of the cinnamic acid derivatives herein studied. To get more insights into the possible mechanism of action of these compounds, the predicted complexes of compound **8** with them were analyzed in detail. In Figure 4 are represented the predicted binding modes of compound **8** to HDAC2, CCC2, CA and CCC3 as well as the diagrams of observed ligand-receptor interactions along the 200 MD snapshots used in MM-PBSA calculations. The binding poses represented in Figure 4 correspond to the centroids of the most populated clusters derived from the clustering of the 200 MD snapshots used for MM-PBSA calculations. All analyses presented below base on the interactions observed in at least 50% of the extracted MD snapshots.

A feature common to all complexes depicted in Figure 4 is the extensive network of contacts between the ligand and the receptors. Among the most probable targets of compound **8**, two are metalloenzymes (CA and HDAC2) and two are ion channels (CCC2 and CCC3). In both metalloenzymes the carbonyl group of the compound points to the Zn^2+^ ions, an interaction that produces favorable electrostatic interactions that positively contribute to the stability of these complexes. The complex of compound **8** with CA is mainly stabilized, in addition to the electrostatic interaction with Zn^2+^, by Van der Waals and hydrophobic interactions. Its phenyl moiety occupies a mostly hydrophobic region lined by W5, Y7, Y66, C67, H97 and T202 while its pentyl tail binds to a zone delimited by H120, V122, V144, L200, T201, V209 and W211.

The predicted binding pose of the ligand to HDAC2 shows the ligand’s aromatic ring stacked between H139, F203 and P204 at the entrance of the binding cavity. On the other hand, the aliphatic part of compound **8** orientates to the bottom of the receptor pocket and interacts with M28, G136, L137, G147, C149, G298, G299 and Y301. As in the complex with CA, compound **8** forms no hydrogen bond with the receptor and most interactions are of hydrophobic and Van der Waals types.

The predicted binding modes of compound **8** to the CCC2 and CCC3 ions transporters are very similar. In both cases the phenyl ring of the ligand orientates toward the bottom of the ions binding cavity while its pentyl moiety heads to the entrance of the pocket. The root mean square deviation (RMSD) between the conformations adopted by compound **8** in both receptors is 2.59 Å. The highly similar binding modes of the ligand to these ion channels can be a consequence of the 65% overall identity between their transmembrane domains and, specifically, of the 79% identity among the residues directly interacting with compound **8**. 

The regions of the binding cavities accommodating the phenyl substituent of compound **8** are formed by I157, G159, V160, M161, N241, P361, S362 and T465 in CCC2 and by the corresponding I137, G139, V140, M141, M220, N221, P341, S342 and T447 residues in CCC3. On the other side of the cavity, the aliphatic moiety of the ligand interacts with F230, N234, T364, G365, Q367, A368, N469 and Y533; and with N136, F210, N214, N217, T344, S348, N451 and F515 in CCC2 and CCC3, respectively. In addition, compound **8** is predicted to hydrogen bond the side chains of N469 and Q237 of CCC2 through its carbonyl group and the main chain oxygen, respectively. The first of these hydrogen bonds is observed in more than 97% of the analyzed MD snapshots while the second one is less frequent (22% of the analyzed snapshots). In contrast, the network of hydrogen bonds predicted for CCC3 is more extensive and it includes the side chains of N217, N451 and N221. All these three residues can serve as hydrogen bond donors for the carbonyl group of compound **8**, while the main chain oxygen of the ligand can accept a hydrogen bond from N217. Overall N217, N451 and N221 of CCC3 are observed to hydrogen bond the ligand in 82%, 73% and 42% of the analyzed MD snapshots, respectively.

The slightly better ΔG of binding predicted for CCC3 compared to CCC2, despite the high identity of their binding pockets, can be explained from the different networks of hydrogen bonds that they form with compound **8**. This difference arises from the N217 (CCC3) to Q237 (CCC2) mutation. In both receptors several hydrogen bonds are formed between the residues pointing to the binding cavity. One of these is formed between the side chains of N241 and Q237 of CCC2, mediated by their amine and carbonyl groups, respectively. The same hydrogen bond is observed between N221 and N217 of CCC3. Notably, these hydrogen bonds are observed during all the 200 MD snapshots analyzed in each system and are not disrupted by the ligand. The side chain of glutamine is one carbon longer than that of asparagine, making the amine and carboxyl groups of the glutamine side chain locating furthest from the protein backbone than in asparagine. This leads to the movement of the side chain of N241 in CCC2, pulled by the hydrogen bond with Q237, to a position unfavorable for hydrogen bonding the carbonyl group of compound **8**. This is not the case of CCC3, where the hydrogen bond between the side chains of the corresponding N221 and N217 favors the formation of hydrogen bonds between them and the ligand.

All these results suggest a multi-target mechanism of action of the cinnamic acid derivatives herein studied. This hypothesis is supported by literature reports where cinnamic acid has been evaluated for its larvicidal activity against *Ae. aegypti*. For instance, the low and moderate ΔGs of binding predicted for GSTD2, GSTT1, GSTX2, GSTE4 and GSTT2 agree with the inhibitory activity of the glutathione s-transferase activity produced by cinnamic acid results reported by Nobsathian and collaborators (2018), [31].

On the light of our results, we postulate that the principal targets of the cinnamic acid derivatives are CA, HDAC2, CCC2 and CCC3. 

Histone deacetylase activity is related to maintaining the stability of the chromosomes in the cell and in proteins expression. However, we found no study in the scientific literature focusing on the larvicidal effect of histone deacetylase inhibitors. On the other hand, carbonic anhydrases has been explored as the targets of *Ae. aegypti* larvicidal compounds [37]. Specifically, CA has been proposed to play an important role in bicarbonate metabolism, pH regulation and ions transportation in the alimentary canal of *Ae. aegypti* [38]. Thus, its inhibition is a strategy to explore for the development of larvicidal compounds.

CCC2 and CCC3 have been characterized as Na^+^-coupled cation chloride cotransporters (CCCs) and associated to ions transport [39,40]. However, the exact mechanisms by which this process occurs remains unclear. It will require more investigations to test the hypothesis that the studied cinnamic acid derivatives could exert their larvicidal effect through the blockage of CCC2 and CCC3. However, we consider that this particular mechanism of action deserves additional attention in future research efforts.

## 3. Materials and Methods 

### 3.1. Chemical Characterization and Reagents

All of the chemical products used during synthesis were from Sigma-Aldrich. ^1^H-NMR (200 and 50 MHz), (400 and 100 MHz) and ^13^C-NMR (500 and 125 MHz) spectra were respectively recorded on VARIAN MERCURY, BRUKER-ASCEND and VARIAN-RMN-SYSTEM spectrometers. Chemical shifts (δ) are expressed in parts per million (ppm) using TMS as an internal standard. Spin multiplicities are given as *s* (singlet), *brs* (broad singlet), *d* (doublet), *t* (triplet), *q* (quartet), *quint* (quintet), *sex* (sextet), *sept* (septet) and *m* (multiplet). High Resolution Mass Spectrometry was performed using an Ultraflex II TOF/TOF spectrometer with a high performance solid state laser (λ = 355 nm), and a reflector using the MALDI technique—matrix assisted laser desorption ionization. Column adsorption chromatography (CC) was performed on silica gel (Merck 60, 230–400 mesh); analytical TLC was performed on pre-coated silica gel plates (Merck 60 F_254_). FTIR spectra were recorded in a Bruker FTIR spectrometer, Vertex 70 model, using KBr pellets.

### 3.2. Synthesis of Compounds ***2**–**9***; Fischer’s Esterification 

To a 100 mL flask, cinnamic acid (0.25 g, 1.69 mmol) and alcohol (50 mL) were added in the presence of sulfuric acid (0.4 mL), and were heated under reflux until completion of the reaction (5–24 h), being verified by single spot TLC. The alcohol was then removed under reduced pressure until about half and the solution was diluted with 20 mL of water. The product was extracted using ethyl acetate (3 × 15 mL), and the organic phase was neutralized successively with 5% sodium bicarbonate, washed with water, then dried over anhydrous sodium sulfate, and filtered. After evaporation under reduced pressure, certain compounds required a column for purification, this phase yielded the ester derivatives, shown in Scheme 1 [41].

### 3.3. Synthesis of Compound ***10***; Reaction with Halide

A mixture of cinnamic acid (0.2 g, 1.35 mmol) in acetone (16.4 mL) was heated under reflux in the presence of triethylamine (0.73 mL) and halide (1.39 mmol) until a complete reaction (24 h), which was verified by single spot TLC. The solvent was then removed under reduced pressure and the solution was diluted with 20 mL of water. The product was extracted with ethyl acetate (3 × 20 mL). The organic phase was treated with water, dried over anhydrous sodium sulfate, filtered, and concentrated under reduced pressure. The residue was purified on a silica gel column (eluent: hexane-ethyl acetate, 9:1), as shown in Scheme 1 [42].

### 3.4. Synthesis of Compounds ***11**–**18***; the Mitsunobu Reaction 

In a 10 mL flash we added cinnamic acid (0.1 g, 0.68 mmol) and alcohol (0.68 mmol); being dissolved in 2.25 mL tetrahydrofuran. The reaction mixture was stirred under magnetic stirring at 0 °C for about 30 min. Afterwards, diisopropyl azodicarboxylate (0.11 mL, 0.68 mmol) and triphenylphosphine (0.15 g, 0.68 mmol) were added as esterification agents, with continuous stirring at room temperature for about 44–68 h, which was verified by a single spot in TLC. The solvent was removed under reduced pressure and the solution was diluted with 10 mL of water. The product was then extracted with ethyl acetate (3 × 10 mL), and the organic phase was neutralized successively with saturated sodium bicarbonate, washed with brine, dried over anhydrous sodium sulfate, filtered, and evaporated under reduced pressure. The residue was then purified on a silica gel column (eluent: hexane-ethyl acetate, 9:1), as shown in Scheme 1 [43].

### 3.5. Chemical Characterization Compounds ***2**–**18***

*Methyl cinnamate* (**2**): Amber amorphous solid; Yield 81.3% (222.6 mg, 1.37 mmol); m.p.: 33–34 °C (lit. 31.2–32.2 °C, [44]; TLC (hexane); R*_f_* = 0.2; IR ʋmax (KBr, cm^−1^): 3067, 3036, 2945, 1717, 1638, 1578, 1454, 1315, 1171, 772 [45]; ^1^H-NMR (CDCl_3_, 400 MHz): δ_H_ 7.70 (1H; *d*; *J* = 16.0 Hz), 7.53–7.51 (2H; *m*), 7.39–7.37 (3H; *m*), 6.44 (1H; *d*; *J* = 16.0 Hz), 3.80 (3H; *s*); ^13^C-NMR (CDCl_3_, 100 MHz): δ_C_ 51.8, 117.9, 128.2, 129.0, 130.4, 134.5, 145.0, 167.5 [46]. 

*Ethyl cinnamate* (**3**): Yellow oil; Yield 84.9% (252.3 mg, 1.43 mmol); TLC (hexane); R*_f_* = 0.22; IR ʋmax (KBr, cm^−1^): 3061, 3028, 2982, 1712, 1639, 1578, 1450, 1312, 1177, 768 [45]; ^1^H-NMR (CDCl_3_, 400 MHz): δ_H_ 7.69 (1H; *d*; *J* = 16.0 Hz), 7.53–7.51 (2H; *m*), 7.39–7.37 (3H; *m*), 6.44 (1H; *d*; *J* = 16.0 Hz), 4.27 (2H; *q*; *J* = 7.2 Hz), 1.34 (3H; *t*; *J* = 7.2 Hz); ^13^C-NMR (CDCl_3_, 100 MHz,): δ_C_ 14.1, 60.5, 118.3, 128.2, 129.0, 130.3, 134.6, 144.7, 167.1 [44,46,47,48].

*Propyl cinnamate* (**4**): Yellow oil; Yield 90.0% (144.4 mg, 0.76 mmol); TLC (hexane); R*_f_* = 0.28; IR ʋmax (KBr, cm^−1^): 3064, 3032, 2969, 1714, 1639, 1579, 1450, 1313, 1174, 768 [45]; ^1^H-NMR (CDCl_3_, 500 MHz): δ_H_ 7.69 (1H; *d*; *J* = 16.0 Hz), 7.53–7.51 (2H; *m*), 7.38–7.37 (3H; *m*), 6.45 (1H; *d*; *J* = 16.0 Hz), 4.17 (2H; *t*; *J* = 7.0 Hz), 1.73 (2H; *sex*; *J* = 7.0 Hz), 1.00 (3H; *t*; *J* = 7.5 Hz); ^13^C-NMR (CDCl_3_, 125 MHz,): δ_C_ 10.7, 22.3, 66.2, 118.4, 128.1, 129.0, 130.3, 134.6, 144.6, 167.1 [44].

*Isopropyl cinnamate* (**5**): Yellow oil; Yield 86.1% (138.2 mg, 0.73 mmol); TLC (hexane); R*_f_* = 0.34; IR ʋmax (KBr, cm^−1^): 3063, 3030, 2981, 1710, 1639, 1579, 1450, 1309, 1176, 768 [45]; ^1^H-NMR (CDCl_3_, 500 MHz): δ_H_ 7.67 (1H; *d*; *J* = 16.0 Hz), 7.52–7.50 (2H; *m*), 7.37–7.36 (3H, *m*), 6.42 (1H; *d*; *J* = 16.0 Hz), 5.14 (1H; *sept*; *J* = 6.0 Hz), 1.32 (6H; *d*; *J* = 6.0 Hz); ^13^C-NMR (CDCl_3_, 125 MHz,): δ_C_ 22.0, 67.9, 118.9, 128.1, 128.9, 130.2, 134.7, 144.4, 166.6 [44,46].

*Methoxyethyl cinnamate* (**6**): Yellow oil; Yield 50.7% (86.2 mg, 0.42 mmol); TLC (9:1 hexane/EtOAc); R*_f_* = 0.34; IR ʋmax (KBr, cm^−1^): 3063, 3032, 2930, 1715, 1638, 1578, 145, 1312, 1173, 768; ^1^H-NMR (CDCl_3_, 400 MHz): δ_H_ 7.71 (1H; *d*; *J* = 16.0 Hz), 7.52–7.49 (2H; *m*), 7.37–7.36 (3H; *m*), 6.48 (1H; *d*; *J* = 16.0 Hz), 4.36 (2H; *t*; *J* = 4.8 Hz), 3.66 (2H; *t*; *J* = 4.8 Hz), 3.41 (3H; *s*); ^13^C-NMR (CDCl_3_, 100 MHz,): δ_C_ 59.1, 63.6, 70.6, 117.9, 128.2, 128.9, 130.4, 134.4, 145.2, 167.0 [46].

*Butyl cinnamate* (**7**): Yellow oil; Yield 89.3% (153.8 mg, 0.75 mmol); TLC (hexane); R*_f_* = 0.2; IR ʋmax (KBr, cm^−1^): 3063, 3030, 2961, 1714, 1639, 1579, 1451, 1311, 1172, 768 [45]; ^1^H-NMR (CDCl_3_, 400 MHz): δ_H_ 7.69 (1H; *d*; *J* = 16.0 Hz), 7.54–7.51 (2H; *m*), 7.39–7.37 (3H; *m*), 6.44 (1H; *d*; *J* = 16.0 Hz), 4.21 (2H; *t*; *J* = 6.8 Hz), 1.70 (2H; *quint*; *J* = 6.8 Hz), 1.44 (2H; *sex*; *J* = 7.2 Hz), 0.97 (3H; *t*; *J* = 7.2 Hz); ^13^C-NMR (CDCl_3_, 100 MHz,): δ_C_ 13.9, 19.3, 30.9, 64.5, 118.4, 128.1, 128.9, 130.3, 134.6, 144.7, 167.2 [48]. 

*Pentyl cinnamate* (**8**): Yellow oil; Yield 68.9% (126.9 mg, 0.58 mmol); TLC (hexane); R*_f_* = 0.22; IR ʋmax (KBr, cm^−1^): 3065, 3032, 2959, 1714, 1639, 1579, 1450, 1311, 1171, 767; ^1^H-NMR (CDCl_3_, 400 MHz): δ_H_ 7.69 (1H; *d*; *J* = 16.0 Hz), 7.54–7.51 (2H; *m*), 7.38–7.37 (3H; *m*), 6.44 (1H; *d*; *J* = 16.0 Hz), 4.20 (2H; *t*; *J* = 6.8 Hz), 1.71 (2H; *quint*; *J* = 6.8 Hz), 1.41–1.36 (4H; *m*), 0.93 (3H; *t*; *J* = 7.2 Hz); ^13^C-NMR (CDCl_3_, 100 MHz,): δ_C_ 14.1, 22.4, 28.2, 28.5, 64.8, 118.4, 128.1, 129.0, 130.3, 134.5, 144.6, 167.2 [46].

*Isopentyl cinnamate* (**9**): Yellow oil; Yield 76.1% (140.2 mg, 0.64 mmol); TLC (hexane); R*_f_* = 0.2; IR ʋmax (KBr, cm^−1^): 3064, 3034, 2960, 1714, 1639, 1579, 1451, 1311, 1168, 767; ^1^H-NMR (CDCl_3_, 400 MHz): δ_H_.7.68 (1H; *d*; *J* = 16.0 Hz), 7.53–7.51 (2H; *m*), 7.38–7.36 (3H; *m*), 6.44 (1H; *d*; *J* = 16.0 Hz), 4.24 (2H; *t*; *J* = 6.8 Hz), 1.80–1.70 (1H; *m*), 1.60 (2H; *q*; *J* = 6.8 Hz), 0.96 (3H; *d*; *J* = 6.4 Hz); ^13^C-NMR (CDCl_3_, 100 MHz,): δ_C_ 22.6, 25.2, 37.6, 63.3, 118.3, 128.1, 129.0, 130.2, 134.6, 144.6, 167.2 [44].

*Hexyl cinnamate* (**10**): Yellow oil; Yield 57.7% (90.4 mg, 0.39 mmol); TLC (hexane); R*_f_* = 0.26; IR ʋmax (KBr, cm^−1^): 3064, 3034, 2958, 1715, 1639, 1579, 1451, 1311, 1171, 768; ^1^H-NMR (CDCl_3_, 400 MHz): δ_H_ 7.69 (1H; *d*; *J* = 16.0 Hz), 7.54–7.52 (2H; *m*), 7.39–7.37 (3H; *m*), 6.45 (1H; *d*; *J* = 16.0 Hz), 4.20 (2H; *t*; *J* = 6.8 Hz), 1.70 (2H; *quint*; *J* = 6.8 Hz), 1.35–1.33 (6H; *m*), 0.91 (3H; *t*; *J* = 7.2 Hz); ^13^C-NMR (CDCl_3_, 100 MHz,): δ_C_ 14.1, 22.7, 25.8, 28.8, 31.6, 64.9, 118.5, 128.2, 129.0, 130.3, 134.6, 144.7, 167.2 [44].

*Dodecyl cinnamate* (**11**): Yellow oil; Yield 48.0% (102.5 mg, 0.32 mmol); TLC (hexane); R*_f_* = 0.26; IR ʋmax (KBr, cm^−1^): 3063, 3028, 2926, 1715, 1639, 1579, 1451, 1310, 1169, 767; ^1^H-NMR (CDCl_3_, 400 MHz): δ_H_ 7.68 (1H; *d*; *J* = 16.0 Hz), 7.54–7.51 (2H; *m*), 7.39–7.37 (3H; *m*), 6.44 (1H; *d*; *J* = 16.0 Hz), 4.20 (2H; *t*; *J* = 6.8 Hz), 1.70 (2H; *quint*; *J* = 6.8 Hz), 1.26 (18H; *brs*), 0.88 (3H; *t*; *J* = 6.8 Hz); ^13^C-NMR (CDCl_3_, 100 MHz,): δ_C_ 14.3, 22.8, 26.1, 28.9, 29.4, 29.5, 29.7, 29.7, 29.8, 29.8, 32.1, 64.9, 118.5, 128.1, 129.0, 130.3, 134.6, 144.6, 167.2 [46].

*4-Chlorobenzyl cinnamate* (**12**): White solid; Yield 43.1% (158.6 mg, 0.58 mmol); m.p.: 62–63 °C (lit. 59.8–60.8 °C, [44]); TLC (9:1 hexane/EtOAc); R*_f_* = 0.48; IR ʋmax (KBr, cm^−1^): 3065, 024, 2963, 1710, 1639, 1594, 1446, 1312, 1163, 1012, 801, 770; ^1^H-NMR (CDCl_3_, 400 MHz): δ_H_ 7.73 (1H; *d*; *J* = 16.0 Hz), 7.54–7.51 (2H; *m*), 7.40–7.38 (3H, *m*), 7.36 (4H; *brs*), 6.48 (1H; *d*; *J* = 16.0 Hz), 5.22 (2H; *s*); ^13^C-NMR (CDCl_3_, 100 MHz,): δ_C_ 65.5, 117.7, 128.2, 128.9, 129.0, 129.8, 130.6, 134.3, 134.4, 134,7, 145.5, 166.8 [46].

*Benzyl cinnamate* (**13**): Yellow oil; Yield 52.9% (85.1 mg, 0.36 mmol); TLC (hexane); R*_f_* = 0.3; IR ʋmax (KBr, cm^−1^): 3066, 3032, 2921, 1717, 1637, 1578, 1450, 1309, 1163, 767 [45]; ^1^H-NMR (CDCl_3_, 400 MHz): δ_H_ 7.75 (1H; *d*; *J* = 16.0 Hz), 7.54–7.53 (2H; *m*), 7.43–7.38 (8H; *m*), 6.51 (1H; *d*; *J* = 16.0 Hz), 5.27 (2H; *s*); ^13^C-NMR (CDCl_3_, 100 MHz,): δ_C_ 66.4, 118.0, 128.2, 128.4, 128.4, 128.7, 129.0, 130.4, 134.5, 136.2, 145.3, 166.9 [44,46,49,50].

*4-Methylbenzyl cinnamate* (**14**): White solid; Yield 63.8% (108.6 mg, 0.43 mmol); m.p.: 41–42 °C; TLC (9:1 hexane/EtOAc); R*_f_* = 0.52; IR ʋmax (KBr, cm^−1^): 3051, 3027, 2922, 1704, 1640, 1574, 1448, 1310, 1187, 803, 768; ^1^H-NMR (CDCl_3_, 400 MHz): δ_H_ 7.73 (1H; *d*; *J* = 16.0 Hz), 7.53–7.51 (2H; *m*), 7.39–7.38 (3H; *m*), 7.33 (2H; *d*; *J* = 8.0 Hz), 7.20 (2H; *d*; *J* = 8.0 Hz), 6.48 (1H; *d*; *J* = 16.0 Hz), 5.22 (2H; *s*), 2.35 (3H; *s*); ^13^C-NMR (CDCl_3_, 100 MHz,): δ_C_ 21.4, 66.4, 118.1, 128.2, 128.6, 129.0, 129.4, 130.4, 133.2, 134.5, 138.3, 145.2, 167.0 [51].

*4-Isopropylbenzyl cinnamate* (**15**): Yellow oil; Yield 52.4% (99.1 mg, 0.35 mmol); TLC (hexane); R*_f_* = 0.22; IR ʋmax (KBr, cm^−1^): 3062, 3029, 2961, 1714, 1638, 1578, 1450, 1310, 1163, 819, 767; ^1^H-NMR (CDCl_3_, 400 MHz): δ_H_ 7,74 (1H; *d*; *J* = 16.0 Hz), 7.54–7.51 (2H; *m*), 7.39–7.36 (5H; *m*), 7.26 (2H; *d*; *J* = 8.0 Hz), 6.49 (1H; *d*; *J* = 16.0 Hz), 5.23 (2H; *s*), 2.94 (1H, *sept*; *J* = 7.2 Hz), 1.27 (6H; *d*; *J* = 7.2 Hz); ^13^C-NMR (CDCl_3_, 100 MHz,): δ_C_ 24.1, 34.0, 66.5, 118.1, 126.8, 128.2, 128.7, 129.0, 130.4, 133.4, 134.5, 145.2, 149.2, 167.0 [51]; HRMS (MALDI) calculated for C_19_H_20_O_2_ [M + Na]^+^: 303.1355, found 303.1365.

*4-Nitrobenzyl cinnamate* (**16**): White crystal solid; Yield 62.0% (118.6 mg, 0.42 mmol); m.p.: 116–117 °C (lit. 117 °C, [49]); TLC (9:1 hexane/EtOAc); R*_f_* = 0.28; IR ʋmax (KBr, cm^−1^): 3083, 3067, 2968, 1709, 1632, 1606, 1450, 1517, 1345, 1312, 1158, 859, 749; ^1^H-NMR (CDCl_3_, 200 MHz): δ_H_ 8.24 (2H; *d*; *J* = 8.0 Hz), 7.76 (1H; *d*; *J* = 16.0 Hz), 7.59–7.55 (4H; *m*), 7.41–7.40 (3H; *m*), 6.51 (1H; *d*; *J* = 16.0 Hz), 5.34 (2H; *s*); ^13^C-NMR (CDCl_3_, 50 MHz,): δ_C_ 64.91, 117.15, 123.93, 128.31, 128.47, 129.08, 130.78, 134.16, 143.52, 146.15, 147.78, 166.55 [49].

*4-Methoxybenzyl cinnamate* (**17**): White solid; Yield 64.8% (117.3 mg, 0.44 mmol); m.p.: 61–62 °C (lit. 59.4–60.9 °C, [44]); TLC (hexane); R*_f_* = 0.22; IR ʋmax (KBr, cm^−1^): 3051, 3035, 2957, 1702, 1643, 1610, 1452, 1249, 1176, 825, 770; ^1^H-NMR (CDCl_3_, 400 MHz): δ_H_ 7.72 (1H; *d*; *J* = 16.0 Hz), 7.53–7.50 (2H; *m*), 7.39–7.36 (5H; *m*), 6.92 (2H; *d*; *J* = 8.0 Hz), 6.47 (1H; *d*; *J* = 16.0 Hz), 5.19 (2H; *s*), 3.82 (3H; *s*); ^13^C-NMR (CDCl_3_, 100 MHz,): δ_C_ 55.4, 66.3, 114.1, 118.1, 128.2, 128.3, 129.0, 130.3, 130.4, 134.5, 145.1, 159.8, 167.0 [44].

*3-Methoxybenzyl cinnamate* (**18**): Yellow oil; Yield 41.5% (75.2 mg, 0.28 mmol); TLC (9:1 hexane/EtOAc); R*_f_* = 0.42; IR ʋmax (KBr, cm^−1^): 3061, 3032, 2939, 1713, 1638, 1605, 1451, 1269, 1166, 865, 768, 685; ^1^H-NMR (CDCl_3_, 400 MHz): δ_H_ 7.74 (1H; *d*; *J* = 16.0 Hz), 7.54–7.51 (2H; *m*), 7.39–7.38 (3H; *m*), 7.30 (1H; *t*; *J* = 7.6 Hz), 7.00 (1H; *dd*; *J* = 2.4 Hz, 8.4 Hz), 6.97–6.96 (1H; *m*), 6.89 (1H; *dd*; *J* = 2.8 Hz, 8.4 Hz), 6.50 (1H; *d*; *J* = 16.0 Hz), 5.23 (2H; *s*), 3.83 (3H; *s*); ^13^C-NMR (CDCl_3_, 100 MHz,): δ_C_ 55.4, 66.4, 113.8, 113.9, 118.0, 120.6, 128.3, 129.0, 129.8, 130.5, 134.5, 137.7, 145.4, 159.9, 166.8 [44].

### 3.6. Effect of Cinnamic Acid Derivatives on Ae. aegypti Larvae

The larvicidal activity of the compounds (**1**–**18**) was evaluated in accordance with the recommendations of the World Health Organization [52]. Fourth-stage *Ae. aegypti* larvae (L4) (Rockefeller strain) were obtained from the Laboratory of Biotechnology Applied to Parasites and Vectors, Biotechnology Center, Federal University of Paraiba. The insects are maintained in a Biological Oxygen Demand (BOD) incubator, under controlled conditions; temperature 27 °C ± 2 °C, relative air humidity 75% ± 5%, and a light and dark 12 h photoperiod.

The compounds were diluted in distilled water (10 mL) at different concentrations (0.0125 to 10 mg/mL). Some substances were diluted in 4% DMSO, while others in 4% Tween80, (due to the compound’s structural characteristics), for later solubilization in distilled water. For a dose response curve and LC_50_ determination, fifteen L4-stage larvae from *Ae. aegypti* were transferred into Falcon tubes containing the 10 mL solution of compounds at different concentrations. The negative control group was prepared using distilled water and a solubilizing agent. The positive control group was prepared using a solution of the insecticides Imiprothrin 0.02%, Permethrin 0.05% and Esbiothrin 0.1% [3]. The tubes were incubated for 24 h at 27 ± 2 °C, over 12 h of natural light and 12 h of darkness [53,54]. Larvae mortality was verified after 24 h of incubation and all tests were carried out in triplicate.

### 3.7. Larvae Mortality Rate 

The larval mortality rate was verified 24 h after exposure to the compounds. The larvae were transferred to petri dishes, being considered lifeless when a total absence of movement was observed. Larvicidal activity was calculated according to Equation (1).

(1) $$Percentage\;of\;larval\;mortality=\frac{Number\;of\;dead\;larvae}{15}\;\times \;100$$

### 3.8. Statistical Analysis

Statistical analysis was performed using GraphPad Prism software for Windows version 5.0 (GraphPad Software, San Diego, CA, USA). Significant differences between groups were analyzed by ANOVA followed by the Tukey post-test when appropriate (*p* < 0.05).

### 3.9. Computational Methods

#### 3.9.1. Molecular Modeling

The overall molecular modeling workflow used in this research bases on that employed in our previous publications [35,36]. The potential molecular targets of the studied series of compounds in *Ae. aegypti* was identified through a consensus computational target fishing approach. Then, the most active compound in the series (**8**) was docked to its potential targets to obtain potential ligand-receptor complexes. The free energies of binding of the compound to its potential targets were estimated from molecular dynamics simulations. Then, the most probable targets of the studied chemicals were selected as those providing the lowest free energies of binding. Figures containing molecular structures were obtained with UCSF Chimera [55], the frequencies of interactions were visualized with Cytoscape [56] and the interaction diagrams were obtained with LigPlot+ 2.2 [57] from the representative MD snapshot. Otherwise noted, the parameters for modeling were kept to their default values in all used software. All molecular modeling steps are described in detail below.

#### 3.9.2. Targets Selection

Compounds **2**, **3**, **5**, **7**, **8** and **13** were independently used as input to the Similarity Ensemble Approach (SEA) web server [58] for the identification of their potential molecular targets. From the lists of predicted targets for these compounds, those predicted for all the six chemicals were selected as potential targets of the congeneric series of compounds. The consensus list of potential targets was used as input to a Blast [59], search, through its NCBI web implementation, against the *Ae. aegypti* (taxid:7159) proteome on the Reference proteins (refseq_protein) database. *Ae. aegypti* proteins identical in at least 40% to any query sequence and covered in at least 80% by the Blast alignment were selected as potential targets of the studied compounds in this organism. Based on literature reports, six glutathione s-transferase and the acetylcholinesterase enzymes of *Ae. aegypti* were also studied as potential targets of the assayed compounds [31,32].

#### 3.9.3. Molecular Docking

The initial three-dimensional structure of compound **8** was obtained with OpenEye’s Omega [60] and partial atomic charges were added to it with Molcharge [61]. For molecular docking, the structure of GSTE2 (code 5FT3) was obtained from the Protein Data Bank [62]. The rest of the evaluated *Ae. aegypti* proteins had no three-dimensional structure solved and homology models were built for them using the SWISS-MODEL web server [63]. Any catalytically relevant cofactor was added to the models of the receptors prior to molecular docking. Furthermore, the binding of compound **8** was evaluated on the glutamate binding site of the GLUR receptor only.

Docking calculations proceeded with the Gold program [64] as previously described [35,36]. Briefly, compound **8** was docked to the selected receptors using the CHEMPL scoring function for primary docking. The search efficiency of Gold was set to 200% and 30 different ligand conformations were predicted for each target. The obtained ligand poses were rescored with the ASP, ChemScore and GoldScore scoring functions of Gold. The obtained binding modes were ranked in descending order according to the consensus score defined by Equation (2):

(2) $$\;Z_{i}=\frac{\sum_{j}\frac{S_{i,j}-\bar{S_{j}}}{std\left(S_{j}\right)}}{4}$$

where, ***S_i_*_,*j*_** is the score of pose *i* according to scoring function *j* and $\bar{S_{j}}$ and ***std*(*S_j_*)** represent the average of scoring function *j* and its standard deviation, respectively. During molecular docking, the side chains of the amino acids directly pointing to the binding cavity were considered flexible. Any predicted pose of compound **8** with a consensus score higher than 1 was selected for further analyses.

#### 3.9.4. Molecular Dynamics Simulations and Free Energies of Binding Estimation

Molecular dynamics (MD) simulations and the estimation of the free energies of binding were carried out with Amber 2018 [65]. The Amber forcefields ff14SB, lipid17 and gaff were used for proteins, lipids and non-aminoacidic residues, respectively. All MD simulations proceeded in explicit solvent.

Compound **8**-receptor complexes were divided into two classes for MD simulations. The first group consisted in water-soluble proteins and the second one was formed by membrane receptors. The later class included the CCC2, CCC3 and KCC1A proteins while the rest of the targets formed the first group. The complexes containing soluble proteins were parameterized with the tleap tool of Amber. These systems were enclosed in truncated octahedron boxes, solvated with TIP3P water molecules and any excess charges in them were neutralized by the addition of either Cl^−^ or Na^+^ ions. Long range electrostatic interactions were treated with the Particle Mesh Ewald (PME) method in all MD simulations steps. The solvated systems underwent a two steps energy minimization protocol, the first of which consisted in 500 steps of the steepest descent method followed by 500 cycles of conjugate gradient at constant volume. During this stage, all atoms except the solvent and counterions were restrained with a force constant of 500 kcal/mol. Å^2^ and the PME distance cutoff was set to 12 Å.

The second part of energy minimization took place at constant volume too and included 1500 iterations of the steepest descent method and 1000 cycles of conjugate gradient, with no restraints and a PME distance cutoff of 10 Å. Afterward, the energy minimized systems were gradually heated from 0 K to 300 K for 20 ps with a time step of 2 fs, keeping all atoms except solvent and counterions restrained with a force constant of 10 kcal/mol.Å^2^. From this step on, the PME distance cutoff was set to 10 Å, all bonds involving hydrogen atoms were constrained with the SHAKE algorithm and temperature was controlled using a Langevin thermostat with a collision frequency of 1.0 ps^−1^.

Next, the systems were equilibrated for 100 ps using a time step of 2 fs at constant pressure and constant temperature. At this step pressure was set to 1 bar and controlled with isotropic position scaling setting the relaxation time to 2 ps. Temperature was set to 300 K. The last snapshot of the equilibration process was used as input to 20 MD production runs. Each of these lasted 2 ns and were initialized with velocities to allow for a better sampling of the complex conformational space.

The complexes of compound **8** with the membrane proteins were prepared with the CHARMM-GUI server [66,67]. These complexes were integrated in a bilayer membrane containing 50 1-palmitoyl−2-oleoylphosphatidylcholine (POPC), 50 1-palmitoyl−2-oleoylphosphatidylethanolamine (POPE) and 25 cholesterol (CHL) molecules on each side. The systems were enclosed in rectangular boxes that were later solvated with TIP3P water molecules and neutralized by adding K^+^ and Cl^−^ counterions. The solvated systems were then energy minimized, heated and equilibrated in Amber using the input files provided by CHARMM-GUI. Production runs were run as for the soluble proteins.

The free energies of binding of compound **8** to all receptors were estimated with the MM-PBSA method as implemented in Amber [67]. MD snapshots for MM-PBSA calculations were extracted from the 40 ns simulations of each target every 200 ps. This lead to 200 snapshots being selected for the free energy of binding calculation on each complex. The ionic strength for these calculations was set to 0.100 mM. Membrane proteins were treated under the implicit membrane model of MMPBSA.py with the membrane dielectric constant set to 4.

## 4. Conclusions

The present study investigated the larvicidal activity of seventeen cinnamic acid esters derivatives against *Ae. aegypti* L. L4 larvae. Based on the findings of the investigation, we concluded that the butyl cinnamate (**7**), pentyl cinnamate (**8**) and benzyl cinnamate (**13**) derivatives present the highest larvicidal activities. The study revealed that certain structural features are important for larvicidal action, such as: alkyl substituents with medium chain; or aryl groups without substituents. Extensive molecular modeling studies on 60 possible protein-inhibitor complexes that involved a total of 2.4 µs MD simulations lead to the proposal of a multi-target mechanism of action for these compounds. Further, the modeling results may guide future experiments focusing on optimizing the larvicidal activity of cinnamic acid derivatives against *Ae. aegypti*, while elucidation their mechanism of action, involving CA, HDAC2, CCC2 and CCC3 proteins, and motivating additional in vitro studies to optimize the larvicidal activity of this class of compounds.

## Figures and Tables

**Table 1:** Larvicidal activity of cinnamic acid and its derivatives against *Ae. aegypti* fourth instar larvae after 24 h of exposure.

Compounds	LC_50_	
	mg/mL	mM
Cinnamic acid (**1**)	0.69 (0.47–0.91)	4.66
Methyl cinnamate (**2**)	0.26 (0.13–0.40)	1.60
Ethyl cinnamate (**3**)	0.13 (0.0–0.32)	0.74
Propyl cinnamate (**4**)	0.53 (0.50–0.56)	2.79
Isopropyl cinnamate (**5**)	0.098 (0.0–0.21)	0.52
Methoxyethyl cinnamate (**6**)	0.12 (0.0–0.33)	0.58
Butyl cinnamate (**7**)	0.042 (0.019–0.091)	0.21
Pentyl cinnamate (**8**)	0.036 (0.0–0.078)	0.17
Isopentyl cinnamate (**9**)	0.18 (0.002–0.36)	0.83
Hexyl cinnamate (**10**)	0.23 (0.0–0.47)	0.99
Dodecyl cinnamate (**11**)	1.12 (1.07–1.17)	3.54
4-Chlorobenzyl cinnamate (**12**)	0.82 (0.62–1.02)	3.01
Benzyl cinnamate (**13**)	0.13 (0.09–0.17)	0.55
4-Methylbenzyl cinnamate (**14**)	3.04 (0.0–8.7)	12.06
4-Isopropylbenzyl cinnamate (**15**)	3.14 (0.76–5.52)	11.21
4-Nitrobenzyl cinnamate (**16**)	3.44 (2.93–3.95)	12.15
4-Methoxybenzyl cinnamate (**17**)	11.42 (0.0–24.43)	42.60
3-Methoxybenzyl cinnamate (**18**)	0.22 (0.0–0.52)	0.82

All means are statistically significant (*p* < 0.05).

**Table 2:** Potential targets of the series of cinnamic acid derivatives in *Ae. aegypti*.

*Ae. aegypti* Target ^a^	Description	VetorBase Gene ^b^	ID
Q174R0	GTPase Rho	AAEL006786	RHO
Q16YG0	Cdc42 homolog	AAEL008543	Cdc42
Q1DH30	Rac GTPase	AAEL015271	RAC
Q16V02	Ras-related protein Rac1	AAEL009733	RAC1
A0A1S4G1K4	Lactoylglutathione lyase	AAEL014393	LGUL
Q16K18	GTPase Rho	AAEL013139	RHO1
Q17BL7	Carbonic anhydrase	AAEL004930	CA
Q17DM5	Aldo-keto reductase	AAEL004088	AKR1
Q17DM8	Aldo-keto reductase	AAEL004102	AKR2
Q17DM9	Aldo-keto reductase	AAEL004118	AKR3
Q17DN2	Aldo-keto reductase	AAEL004086	AKR4
Q17DN0	Aldo-keto reductase	AAEL004095	AKR5
A0A1S4F6Y1	Aldo-keto reductase	AAEL004096	AKR6
Q16QZ8	Histone deacetylase	AAEL011117	HDAC1
Q17CF0	Histone deacetylase	AAEL004586	HDAC2
Q17GT4	Thioredoxin reductase	AAEL002886	TRXR
A0A1S4F1W5	Glutamate receptor, ionotropic kainate 1, 2, 3 (glur5, glur6, glur7)	AAEL002506	GLUR
A0A1S4FNU9	Sodium-coupled cation-chloride cotransporter	AAEL009886	CCC3
A0A1S4FNN9	Sodium-coupled cation-chloride cotransporter	AAEL009888	CCC2
H2L215	SLC12-like K,Cl cotransporter	AAEL019507	KCC1A
Q5PY77	Glutathione transferase	AAEL007951	GSTE2
Q5PY78	Glutathione transferase	AAEL007962	GSTE4
Q17MB0	Glutathione transferase	AAEL026530	GSTD2
Q5PY76	Glutathione transferase	AAEL009017	GSTT1
Q95W09	Glutathione transferase	AAEL010500	GSTX2
Q5PY75	Glutathione transferase	AAEL009016	GSTT2
Q6A2E2	Acetylcholinesterase	AAEL000511	ACE

^a^ UniProt entry name. ^b^ Gene ID in the VectorBase database.

**Table 3:** Results of the molecular docking of compound **8** to its potential targets. Docking scores are dimensionless.

Target	Conformer	CHEMPLP		GoldScore		ChemScore		ASP		Consensus Z-Score
		Score	Z-Score	Score	Z-Score	Score	Z-Score	Score	Z-Score	
RHO	1	58.90	1.77	33.80	1.49	20.58	1.86	21.71	2.19	1.83
	2	60.45	2.22	29.36	0.35	20.12	1.60	19.45	1.27	1.36
	3	58.63	1.69	33.92	1.52	17.82	0.32	18.23	0.78	1.08
Cdc42	1	55.95	1.12	30.55	0.53	20.55	2.09	25.03	1.82	1.39
	2	57.58	1.66	30.47	0.51	18.40	1.00	23.27	1.26	1.11
RAC	1	64.39	1.82	39.30	1.85	20.91	1.78	22.54	1.55	1.75
	2	60.10	0.78	35.55	0.74	20.07	1.42	22.60	1.57	1.13
RAC1	1	64.31	1.35	38.03	1.20	24.44	2.63	27.91	2.80	1.99
	2	64.50	1.40	39.60	1.55	21.82	1.31	23.21	0.99	1.31
	3	62.32	0.83	40.51	1.76	21.89	1.34	23.61	1.14	1.27
LGUL	1	69.59	3.01	28.25	0.49	33.02	2.77	30.47	1.75	2.01
RHO1	1	60.44	1.90	38.37	2.35	20.55	1.36	19.10	1.17	1.70
	2	56.37	1.23	35.55	1.63	18.01	0.76	20.78	1.49	1.28
	3	57.11	1.35	31.61	0.61	22.01	1.71	19.50	1.25	1.23
CA	1	57.40	1.95	43.35	3.48	20.03	1.30	18.68	−0.73	1.50
	2	53.26	0.90	29.63	0.28	21.65	1.93	29.44	2.33	1.36
	3	58.64	2.27	31.04	0.61	21.26	1.77	21.29	0.02	1.17
AKR1	1	59.43	1.59	35.51	0.92	21.82	1.45	33.55	2.17	1.53
	2	58.53	1.32	37.08	1.32	20.79	0.89	29.64	1.03	1.14
AKR2	1	64.38	1.71	36.58	0.49	21.94	1.41	29.62	1.27	1.22
	2	60.40	0.68	39.87	1.27	21.92	1.41	28.09	0.75	1.03
AKR3	1	55.03	1.49	10.39	0.53	23.22	1.42	33.37	2.12	1.39
	2	52.82	1.05	11.38	0.59	22.37	1.12	32.26	1.75	1.13
AKR4	1	62.93	2.13	2.64	0.36	25.43	1.95	25.47	0.22	1.17
AKR5	1	54.97	2.35	30.42	1.12	22.85	2.23	23.56	1.80	1.88
	2	46.66	0.92	31.07	1.28	19.92	1.37	25.33	2.34	1.48
	3	51.13	1.69	28.61	0.68	22.56	2.14	20.01	0.72	1.31
AKR6	1	52.76	1.67	18.87	0.63	18.64	0.69	25.90	2.32	1.33
HDAC1	1	54.25	1.57	33.14	1.85	18.90	1.35	31.50	1.91	1.67
	2	57.87	2.60	30.91	1.23	17.18	0.36	32.16	2.09	1.57
	3	51.85	0.88	30.17	1.02	19.73	1.83	28.16	0.98	1.18
HDAC2	1	67.83	3.02	33.28	1.53	21.64	1.97	34.36	2.65	2.29
	2	66.26	2.59	40.21	3.52	20.00	0.67	33.13	2.23	2.25
TRXR	1	58.20	1.62	33.85	1.91	19.07	0.85	26.92	1.26	1.41
	2	57.65	1.53	27.63	0.35	20.51	1.25	26.64	1.20	1.08
	3	50.72	0.32	33.48	1.82	18.79	0.77	26.62	1.20	1.03
GLUR	1	43.93	1.45	38.20	1.17	9.93	0.41	20.16	1.99	1.25
	2	44.38	1.55	35.82	0.78	12.64	1.01	16.41	1.35	1.17
	3	42.44	1.09	32.79	0.28	14.03	1.31	18.62	1.72	1.10
CCC3	1	42.91	1.25	31.55	0.86	12.49	0.73	18.77	1.51	1.09
CCC2	1	51.24	2.05	42.66	2.29	15.44	1.10	19.54	1.40	1.71
	2	48.60	1.56	34.18	0.85	16.51	1.51	18.79	1.22	1.29
KCC1A	1	59.45	2.89	37.85	2.71	15.65	−1.46	21.44	0.32	1.11
	2	53.34	1.03	28.62	0.71	21.27	0.82	25.40	1.86	1.11
GSTE2	1	63.42	0.91	36.27	1.53	27.20	1.88	24.47	0.60	1.23
GSTE4	1	64.51	1.68	30.14	0.06	25.20	1.79	24.30	1.72	1.31
	2	63.20	1.31	34.97	1.26	24.36	1.43	22.21	0.92	1.23
	3	64.03	1.55	33.48	0.89	22.31	0.53	22.96	1.21	1.04
GSTD2	1	50.10	1.08	31.62	1.81	19.91	1.64	25.27	2.17	1.68
	2	52.29	2.04	25.51	0.02	20.10	1.75	25.09	2.10	1.48
	3	49.98	1.03	32.08	1.95	20.32	1.87	22.45	1.05	1.47
	4	54.75	3.11	29.78	1.27	18.43	0.84	20.16	0.13	1.34
GSTT1	1	46.82	1.27	37.47	2.55	14.90	0.97	13.96	0.24	1.26
	2	45.26	0.94	31.40	1.22	14.95	0.99	17.65	1.39	1.13
GSTX2	1	64.43	2.54	35.83	0.67	22.83	0.35	27.07	1.11	1.17
GSTT2	1	55.10	1.41	35.79	1.03	19.27	1.42	22.70	1.26	1.28
	2	55.08	1.40	36.45	1.17	18.77	1.13	22.13	1.01	1.18
	3	53.66	0.92	35.78	1.02	18.55	1.00	22.90	1.34	1.07
ACE	1	66.49	2.03	38.42	1.51	27.23	1.26	40.77	1.71	1.63
	2	63.40	1.07	35.51	0.71	26.21	0.69	40.82	1.72	1.05

**Table 4:** Estimated free energies of binding (last column) of compound **8** to its potential targets and their components. Energy values are expressed in kcal/mol.

Target	Conformer	MM-PBSA Component							ΔG TOTAL
		VDWAALS	EEL	EPB	ENPOLAR	EDISPER	DELTA G Gas	DELTA G Solv	
RHO	1	−29.83	−10.07	23.41	−23.48	36.28	−39.90	36.20	−3.70
	2	−25.21	−4.55	18.20	−20.40	31.68	−29.76	29.47	−0.29
	3	−18.54	−4.42	14.80	−15.28	25.09	−22.97	24.61	1.64
Cdc42	1	−21.78	−3.06	14.44	−18.36	28.95	−24.84	25.03	0.19
	2	−26.98	−7.22	21.43	−21.01	35.15	−34.20	35.57	1.37
RAC	1	−28.43	−10.29	25.03	−23.27	35.58	−38.72	37.34	−1.38
	2	−31.63	−2.24	20.23	−24.42	37.46	−33.88	33.27	−0.61
RAC1	1	−28.67	−10.89	28.85	−22.23	35.55	−39.55	42.17	2.62
	2	−25.52	−5.29	20.96	−20.64	32.56	−30.81	32.88	2.07
	3	−30.26	−10.77	26.13	−24.38	37.11	−41.03	38.85	−2.18
LGUL	1	−24.40	−25.51	35.21	−24.56	39.18	−49.90	49.83	−0.07
RHO1	1	−25.42	−6.01	19.92	−20.62	32.41	−31.43	31.71	0.28
	2	−23.94	−4.63	17.11	−19.43	31.08	−28.57	28.76	0.19
	3	−26.08	−5.48	18.58	−20.67	32.40	−31.56	30.31	−1.25
CA	1	−31.39	−37.24	35.91	−28.63	43.79	−68.63	51.06	−17.57
	2	−35.19	−10.00	26.43	−27.00	41.71	−45.19	41.14	−4.05
	3	−29.70	−38.59	39.81	−27.36	43.77	−68.29	56.22	−12.07
AKR1	1	−32.60	−7.54	22.94	−25.50	40.65	−40.13	38.09	−2.04
	2	−34.44	−11.13	27.87	−26.01	41.34	−45.57	43.21	−2.36
AKR2	1	−25.62	−4.71	20.90	−20.50	35.55	−30.33	35.95	5.62
	2	−27.29	−3.71	16.04	−21.99	36.13	−30.99	30.17	−0.82
AKR3	1	−26.18	−5.66	18.09	−22.71	36.63	−31.84	32.02	0.17
	2	−32.10	−9.72	21.75	−26.18	40.80	−41.82	36.37	−5.45
AKR4	1	−35.96	−8.29	24.18	−28.56	45.11	−44.25	40.74	−3.51
AKR5	1	−28.66	−3.77	17.40	−23.05	37.38	−32.44	31.74	−0.70
	2	−28.63	−6.59	22.85	−22.50	38.13	−35.22	38.48	3.26
	3	−26.62	−5.04	17.17	−21.58	34.92	−31.66	30.51	−1.15
AKR6	1	−31.54	−8.85	19.78	−26.51	41.95	−40.38	35.21	−5.17
HDAC1	1	−25.06	−2.02	16.86	−19.07	31.83	−27.08	29.63	2.56
	2	−32.98	−8.30	31.81	−24.47	40.25	−41.28	47.59	6.31
	3	−32.33	−12.10	26.99	−23.74	38.74	−44.43	41.98	−2.45
HDAC2	1	−28.79	−47.26	47.24	−26.80	43.29	−76.05	63.73	−12.32
	2	−33.84	−4.59	23.46	−24.07	39.96	−38.43	39.35	0.92
TRXR	1	−28.27	−3.60	18.81	−22.35	36.89	−31.87	33.35	1.47
	2	−26.53	−6.41	20.67	−20.50	34.85	−32.94	35.02	2.07
	3	−23.49	−4.74	21.24	−19.13	32.89	−28.24	35.00	6.77
GLUR	1	−30.46	−3.63	22.92	−24.00	38.87	−34.09	37.79	3.70
	2	−34.17	−7.47	25.09	−26.45	43.36	−41.63	42.00	0.37
	3	−37.26	−9.08	35.26	−27.81	44.66	−46.34	52.10	5.76
CCC3	1	−41.84	−8.25	10.76	−28.87	47.67	−50.09	29.56	−20.53
CCC2	1	−9.06	−4.09	75.89	−30.93	47.72	−13.15	92.68	79.53
	2	−44.26	0.76	10.52	−29.36	46.69	−43.50	27.85	−15.64
KCC1A	1	−38.70	38.29	10.86	−27.73	46.16	−0.41	29.29	28.87
	2	−38.64	3.52	10.86	−27.86	46.84	−35.12	29.84	−5.28
GSTE2	1	−24.34	−1.90	13.17	−20.38	33.97	−26.24	26.76	0.52
GSTE4	1	−30.05	−6.13	20.59	−25.19	40.32	−36.18	35.73	−0.45
	2	−31.98	−2.82	15.71	−25.69	41.17	−34.80	31.19	−3.61
	3	−27.34	−0.81	13.45	−22.34	36.93	−28.15	28.05	−0.10
GSTD2	1	−20.18	−2.36	11.48	−16.94	27.99	−22.55	22.52	−0.02
	2	−17.26	−1.94	9.18	−14.83	24.47	−19.20	18.82	−0.38
	3	−19.71	−1.55	8.90	−16.27	26.90	−21.26	19.54	−1.72
	4	−17.93	−0.89	9.04	−14.96	24.69	−18.82	18.76	−0.06
GSTT1	1	−19.24	−2.73	10.98	−16.64	27.32	−21.97	21.66	−0.31
	2	−25.45	−4.02	14.12	−21.37	34.20	−29.46	26.95	−2.51
GSTX2	1	−29.27	−3.04	15.38	−24.22	37.99	−32.31	29.15	−3.16
GSTT2	1	−29.29	−11.48	23.41	−24.17	36.64	−40.77	35.88	−4.89
	2	−27.99	−7.79	20.40	−23.26	36.31	−35.78	33.45	−2.33
	3	−30.40	−8.32	23.34	−25.04	39.16	−38.72	37.46	−1.26
ACE	1	−37.59	−2.92	55.56	−27.91	45.47	−40.51	73.12	32.61
	2	−36.45	−3.73	29.43	−27.15	45.03	−40.17	47.30	7.13

## Supplementary Materials

The following are available online, Figure S1: Total energy, in kcal/mol, of the studied complexes along the MD simulations, Figure S2: Ligand RMSD, in Å, relative to the starting docking conformation along the MD simulations.

## Sample Availability

Samples of the compounds **1**−**18** are available from authors (or from MDPI).
