# Supplementary material for: Larvicidal Activity of Cinnamic Acid Derivatives: Investigating Alternative Products for Aedes aegypti L. Control
Source: Molecules. 2020 Dec 24;26(1):61. doi: 10.3390/molecules26010061 (PMC7796249; doi:10.3390/molecules26010061)
Supplement: Supplementary file 1 [file molecules-26-00061-s001.pdf]

Supplementary Materials

Larvicidal activity of Cinnamic Acid Derivatives: Investigating Alternative Products for *Aedes aegypti* L. Control.

Marianna O. Araújo [1], Yunierkis Pérez-Castillo [2], Louise H. G. Oliveira [3], Fabíola C. Nunes [3] and Damião P. de Sousa [1,4,*]

[1] Post Graduation Program in Natural and Synthetic Bioactive Products, Federal University of Paraíba, João Pessoa 58051-900, PB, Brazil; marianna.oliveira@ltf.ufpb.br (M.O.A); damiao_desousa@yahoo.com.br (D.P.S)

[2] Bio-Cheminformatics Research Group and Escuela de Ciencias Físicas y Matemáticas, Universidad de Las Américas, Quito 170125, Ecuador; yunierkis.perez@udla.edu.ec

[3] Biotechnology Center, Federal University of Paraíba, João Pessoa 58051-900, PB, Brazil; louiseguimaraes@outlook.com (L.H.G.O.); fabiola@cbiotec.ufpb.br (F.C.N.)

[4] Department of Pharmaceutical Sciences, Federal University of Paraíba, CEP 58051-970, João Pessoa, PB, Brazil

* Correspondence: damiao_desousa@yahoo.com.br; Tel.: +55 833216-7347

Figure S1. Total energy, in kcal/mol, of the studied complexes along the MD simulations. 2

Figure S2. Ligand RMSD, in Å, relative to the starting docking conformation along the MD simulations. 7

Figure S1. Total energy, in kcal/mol, of the studied complexes along the MD simulations.

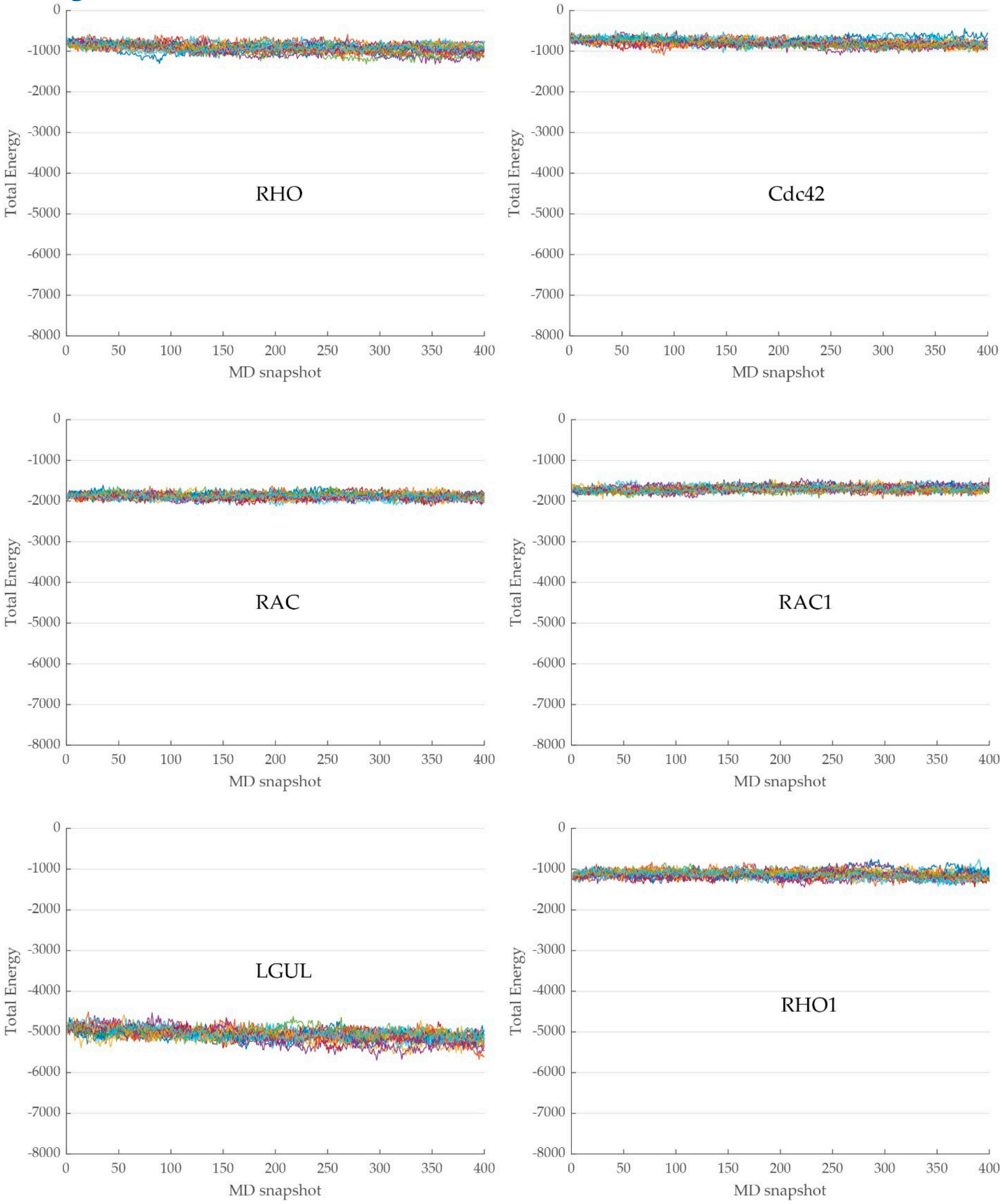

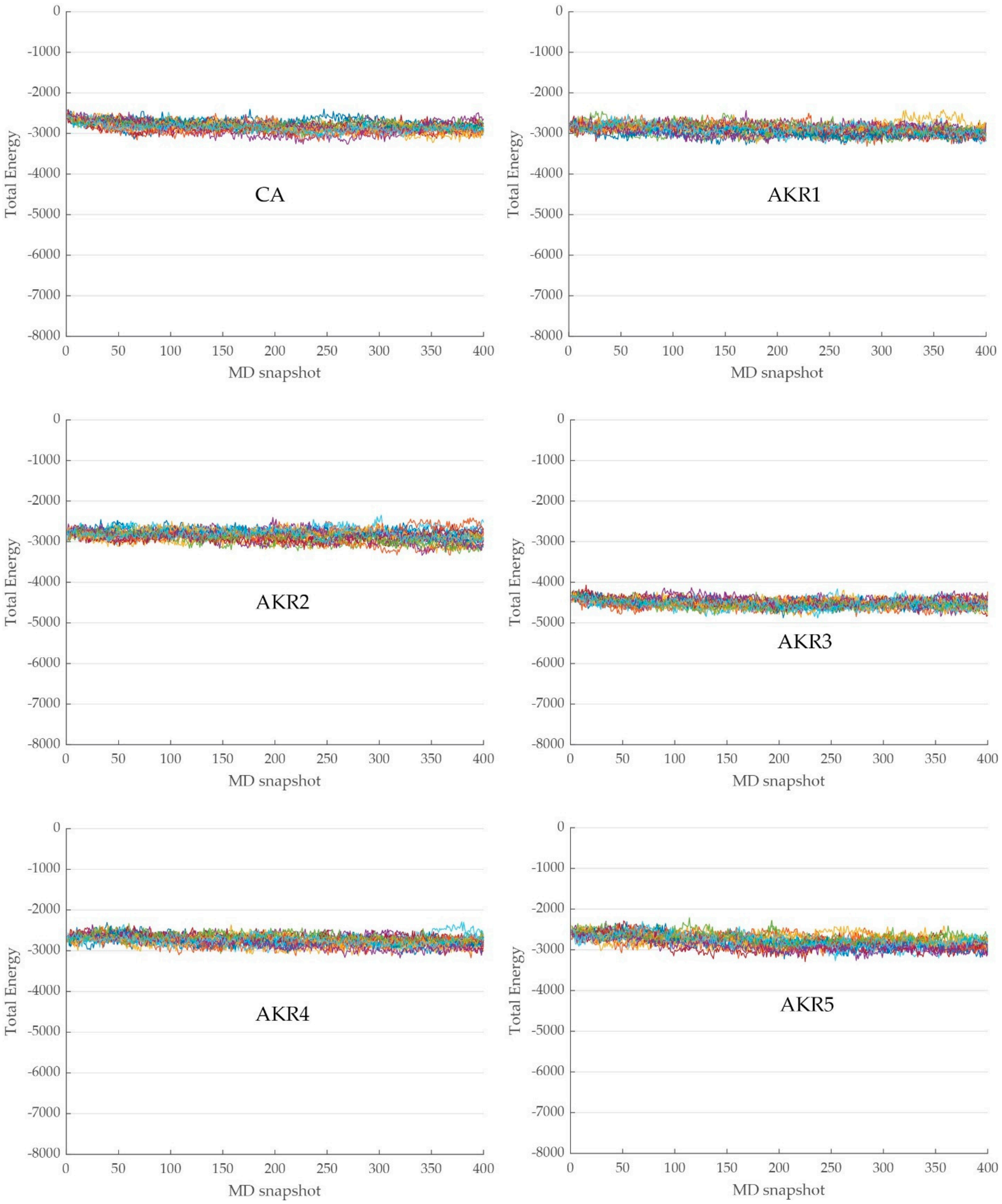
CA
AKR1
AKR2
AKR3
AKR4
AKR5
Total Energy
MD snapshot

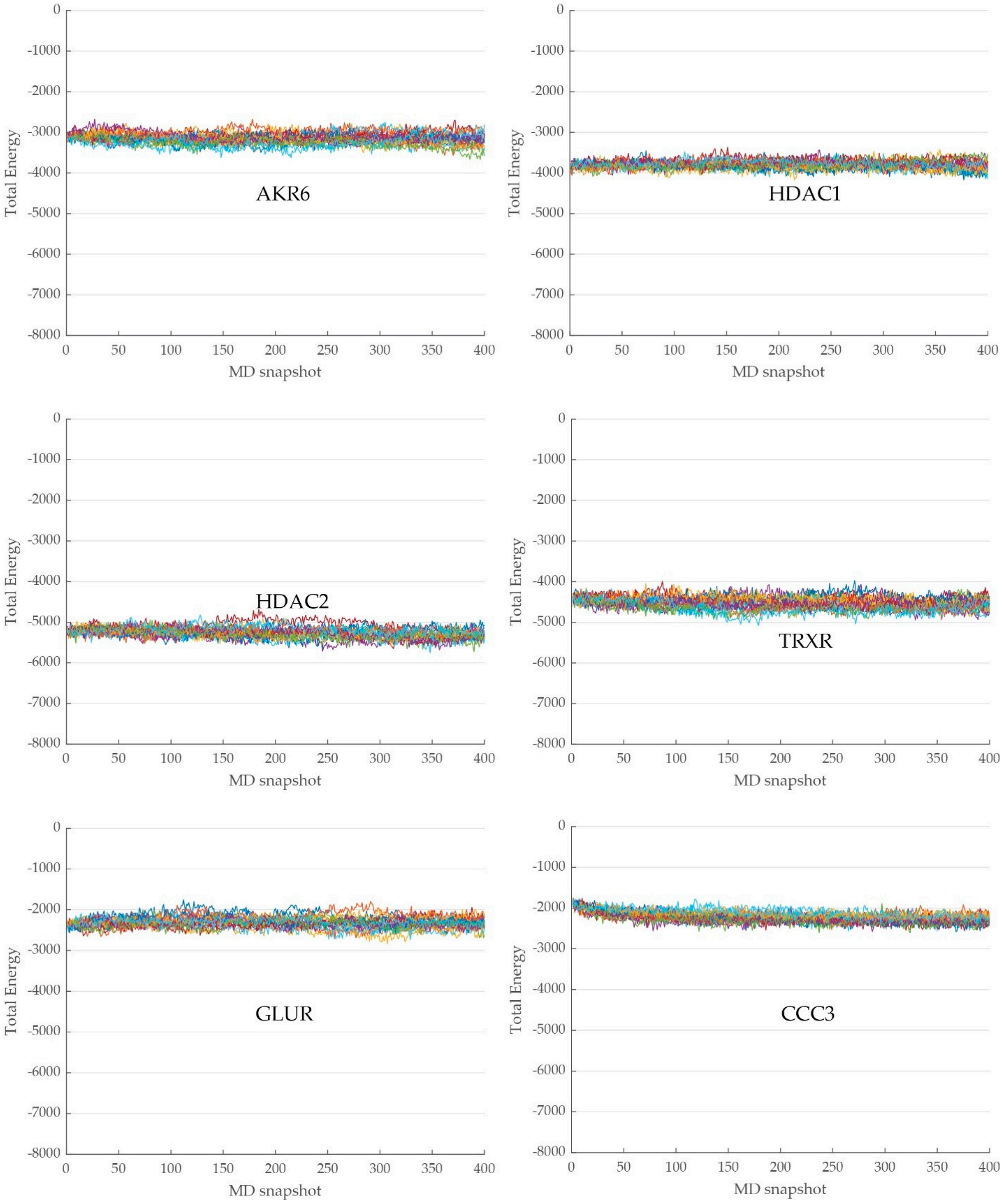
AKR6
HDAC1
HDAC2
TRXR
GLUR
CCC3
Total Energy
MD snapshot

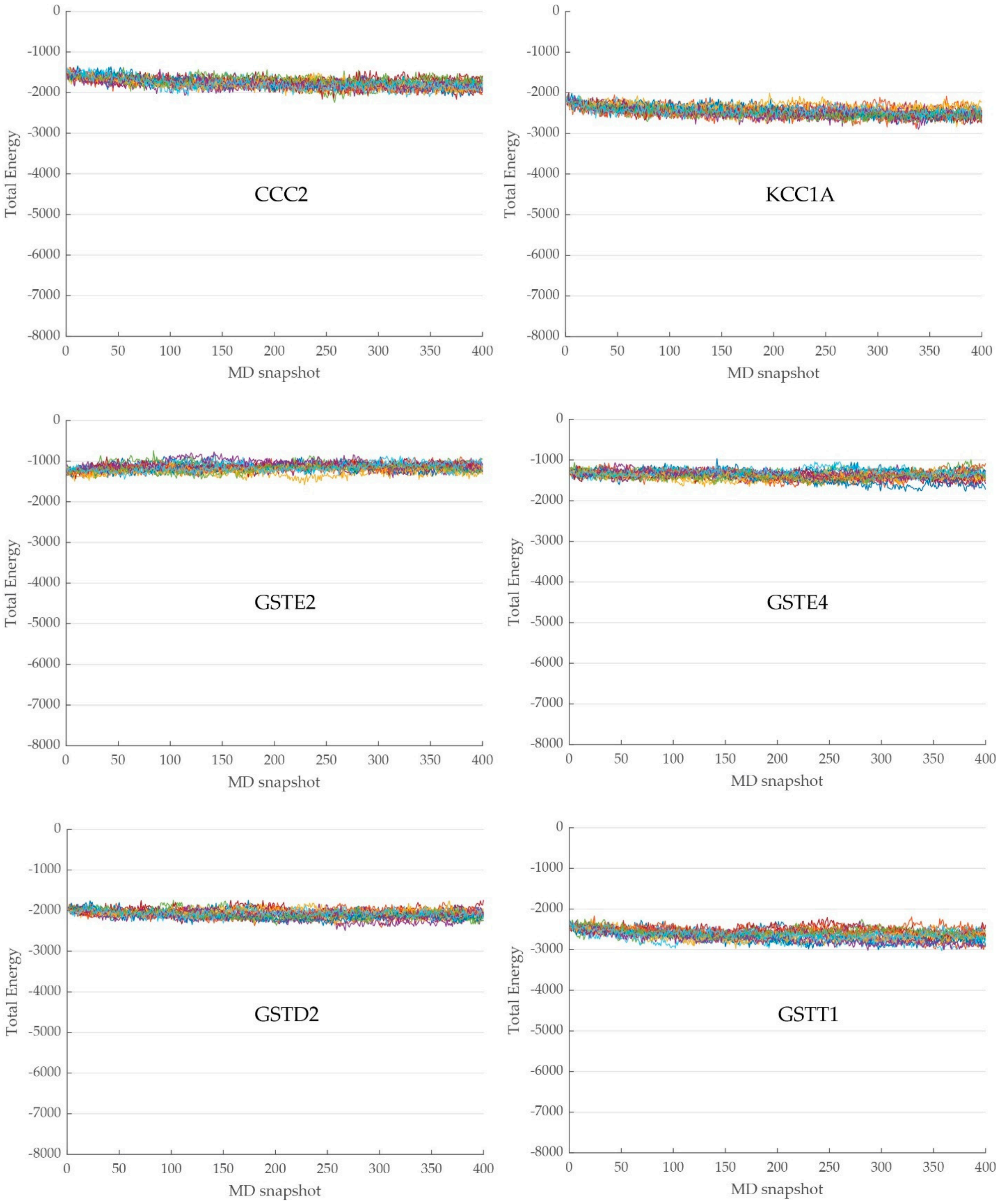

CCC2
KCC1A
GSTE2
GSTE4
GSTD2
GSTT1
Total Energy
MD snapshot
0
-1000
-2000
-3000
-4000
-5000
-6000
-7000
-8000
0 50 100 150 200 250 300 350 400

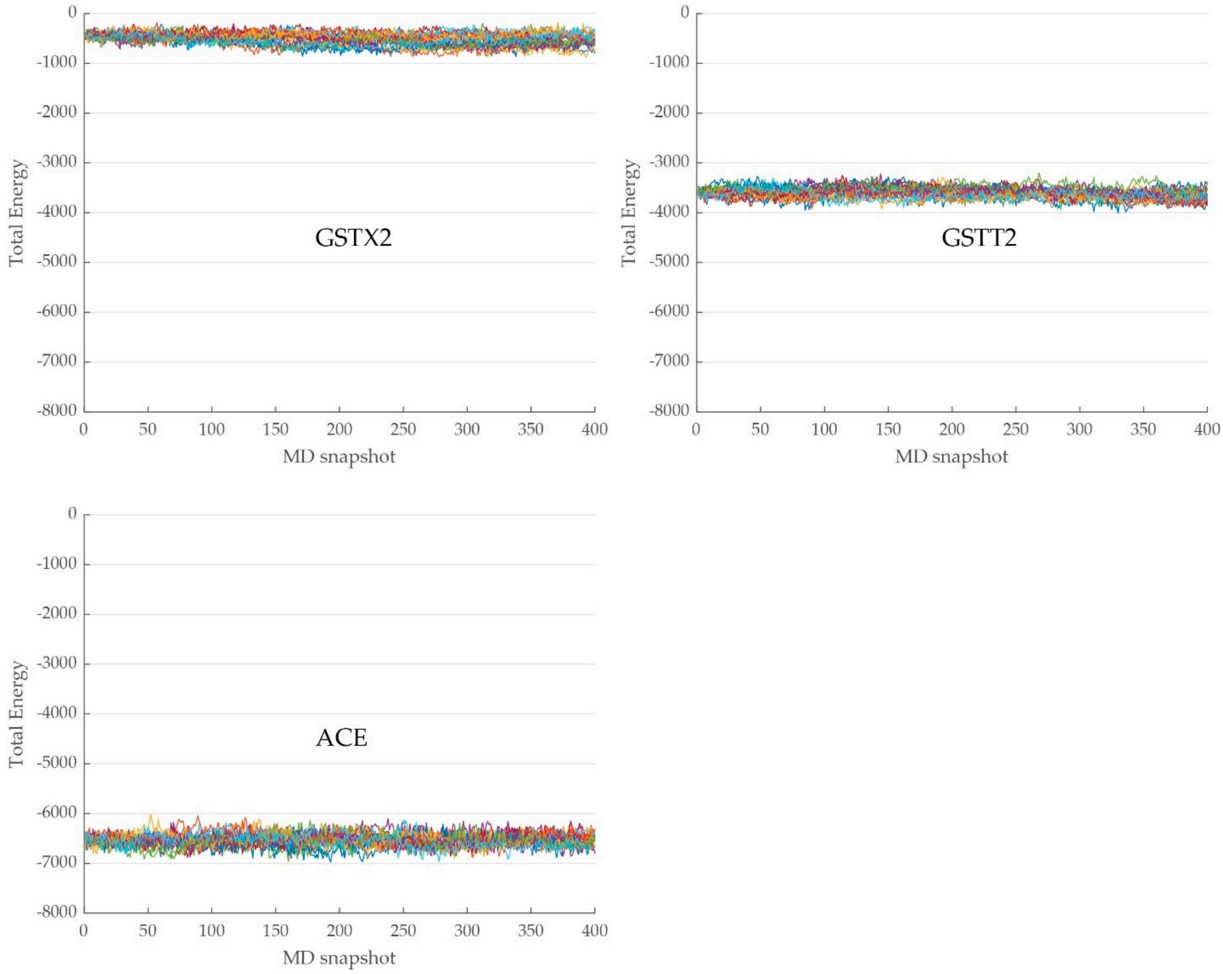
GSTX2
GSTT2
ACE
Total Energy
MD snapshot
0
-1000
-2000
-3000
-4000
-5000
-6000
-7000
-8000
0 50 100 150 200 250 300 350 400

Figure S2. Ligand RMSD, in Å, relative to the starting docking conformation along the MD simulations.

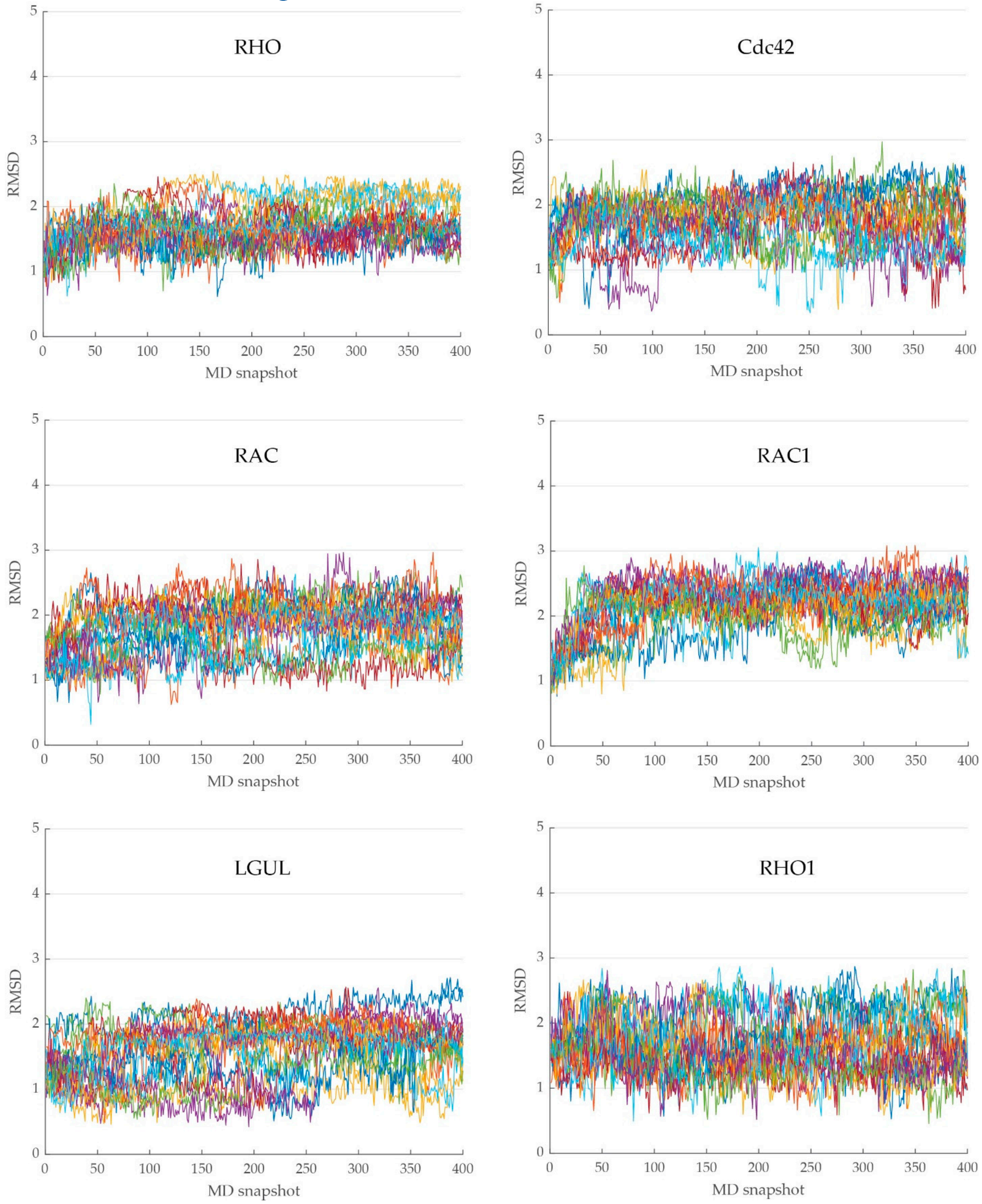

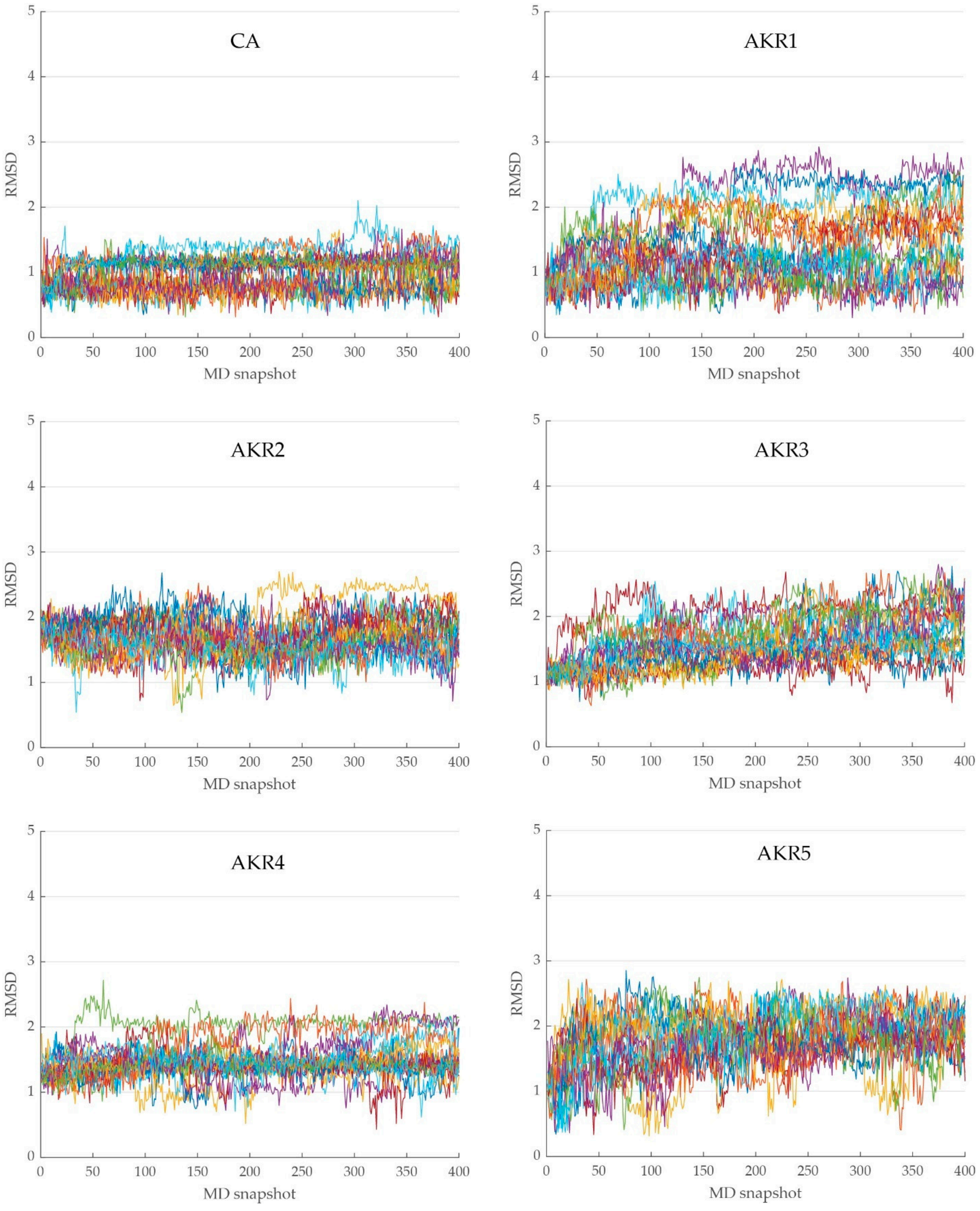
CA
AKR1
AKR2
AKR3
AKR4
AKR5
RMSD
MD snapshot
0
1
2
3
4
5
50
100
150
200
250
300
350
400

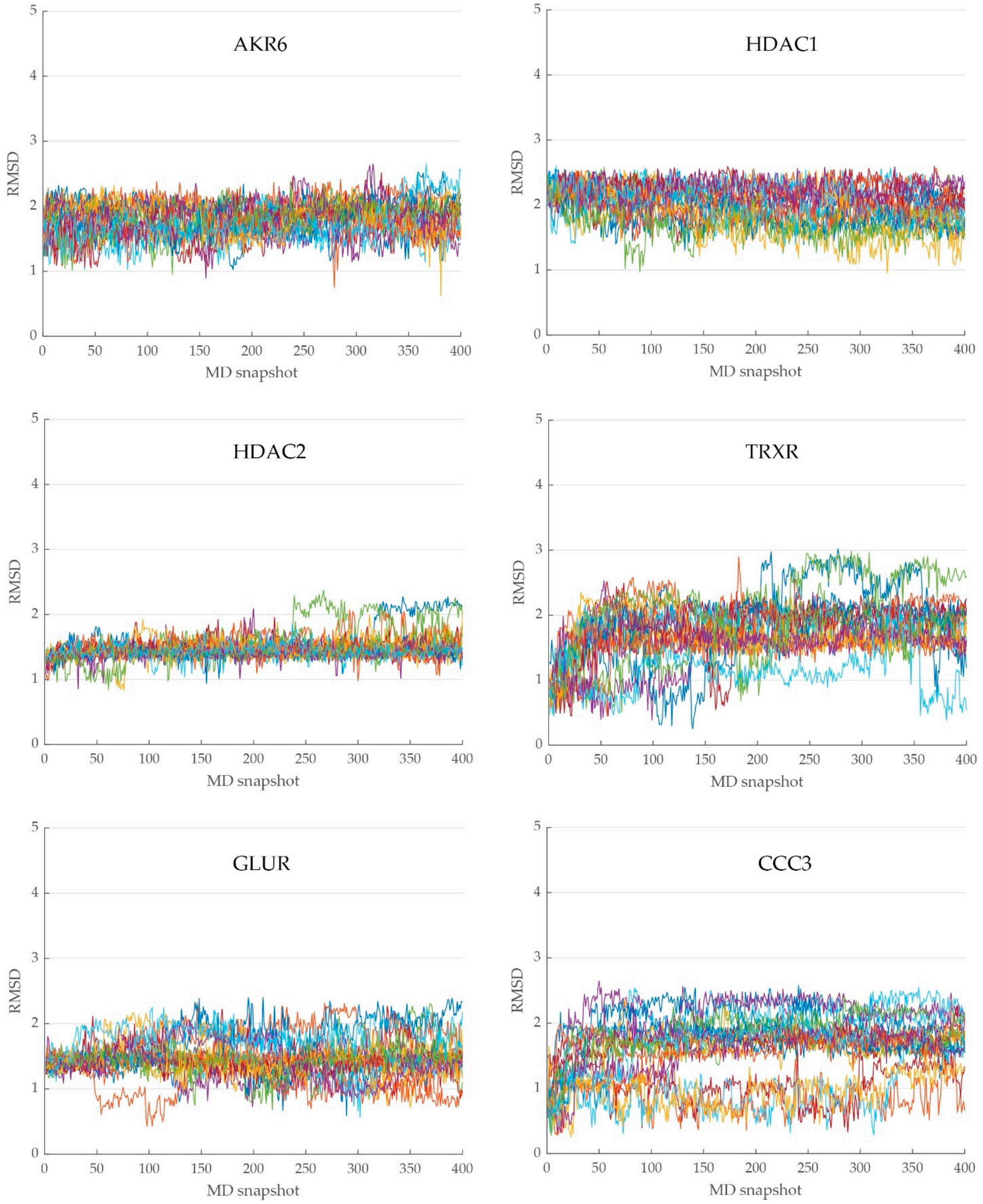
AKR6
HDAC1
HDAC2
TRXR
GLUR
CCC3
RMSD
MD snapshot
0 1 2 3 4 5
0 50 100 150 200 250 300 350 400

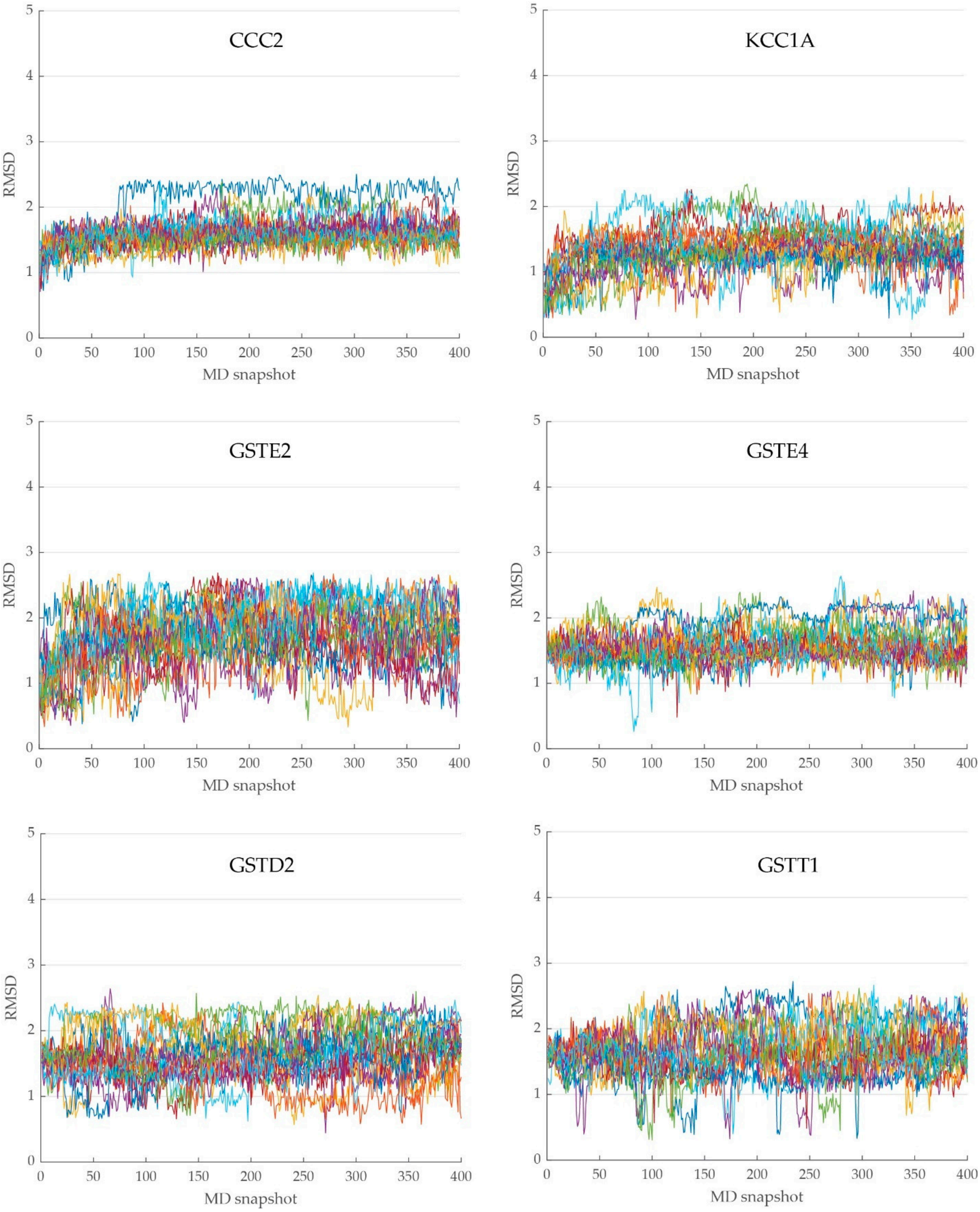
CCC2
KCC1A
GSTE2
GSTE4
GSTD2
GSTT1
RMSD
MD snapshot
0
1
2
3
4
5
0
50
100
150
200
250
300
350
400

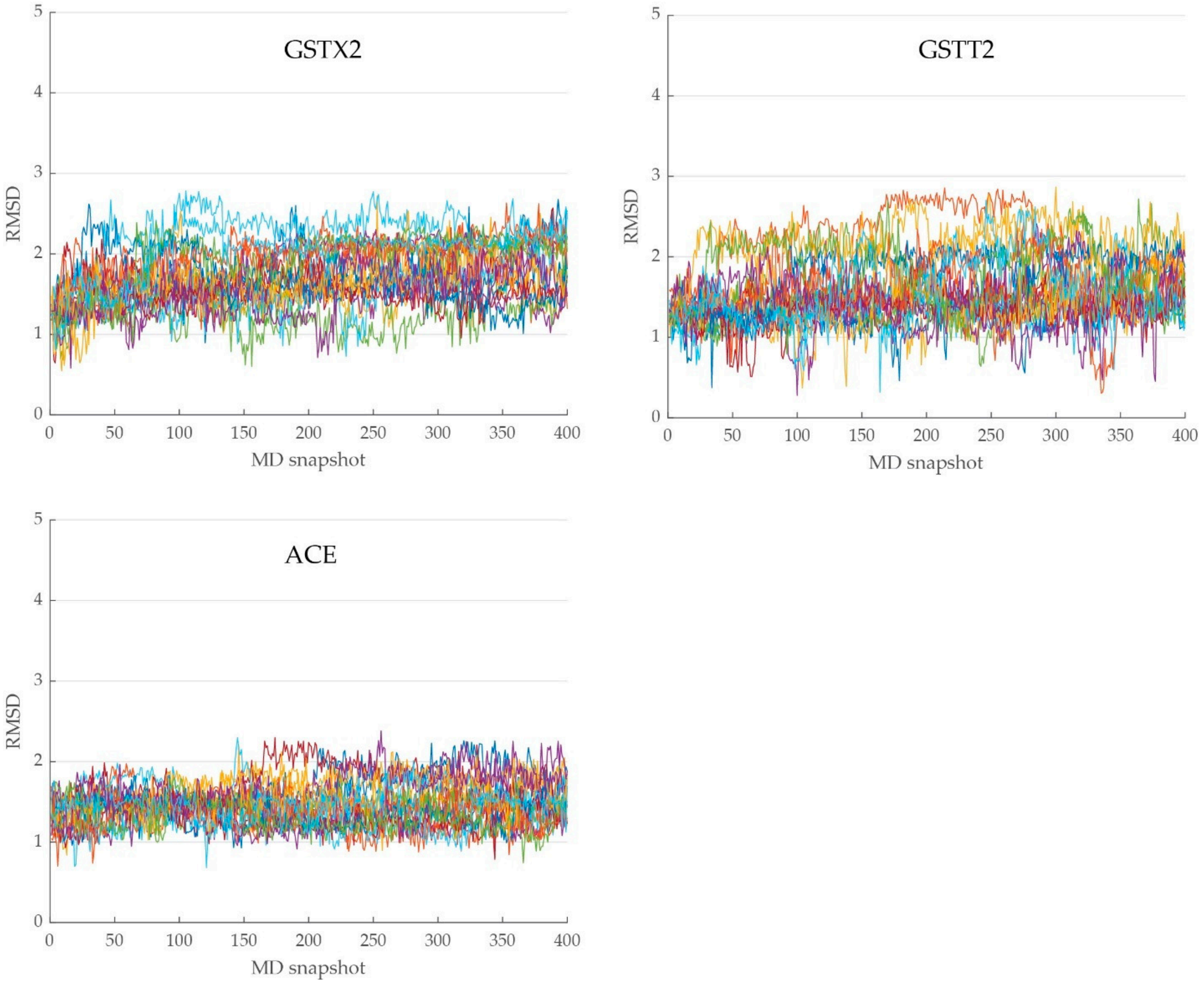
GSTX2
GSTT2
ACE
RMSD
MD snapshot
0
1
2
3
4
5
50
100
150
200
250
300
350
400
